# Fractional-Order Boosted Hybrid Young’s Double-Slit Experimental Optimizer for Truss Topology Engineering Optimization

**DOI:** 10.3390/biomimetics9080474

**Published:** 2024-08-05

**Authors:** Song Qin, Junling Liu, Xiaobo Bai, Gang Hu

**Affiliations:** 1 School of Art and Design, Xi’an University of Technology, Xi’an 710054, China; qinbinbin@126.com (S.Q.); xiaobo413@126.com (X.B.); 2 National Demonstration Center for Experimental Arts Education, Nankai University, Tianjin 300371, China; 3 Department of Applied Mathematics, Xi’an University of Technology, Xi’an 710054, China; hugang@xaut.edu.cn

**Keywords:** Young’s double-slit experiment, fractional-order strategy, piecewise chaotic mapping, dynamic opposition, vertical operator

## Abstract

Inspired by classical experiments that uncovered the inherent properties of light waves, Young’s Double-Slit Experiment (YDSE) optimization algorithm represents a physics-driven meta-heuristic method. Its unique search mechanism and scalability have attracted much attention. However, when facing complex or high-dimensional problems, the YDSE optimizer, although striking a good balance between global and local searches, does not converge as fast as it should and is prone to fall into local optimums, thus limiting its application scope. A fractional-order boosted hybrid YDSE, called FYDSE, is proposed in this article. FYDSE employs a multi-strategy mechanism to jointly address the YDSE problems and enhance its ability to solve complex problems. First, a fractional-order strategy is introduced into the dark edge position update of FYDSE to ensure more efficient use of the search potential of a single neighborhood space while reducing the possibility of trapping in a local best. Second, piecewise chaotic mapping is constructed at the initial stage of the population to obtain better-distributed initial solutions and increase the convergence rate to the optimal position. Moreover, the low exploration space is extended by using a dynamic opposition strategy, which improves the probability of acquisition of a globally optimal solution. Finally, by introducing the vertical operator, FYDSE can better balance global exploration and local exploitation and explore new unknown areas. The numerical results show that FYDSE outperforms YDSE in 11 (91.6%) of cec2022 sets. In addition, FYDSE performs best in 8 (66.6%) among all algorithms. Compared with the 11 methods, FYDSE obtains the optimal best and average weights for the 20-bar, 24-bar, and 72-bar truss problems, which proves its efficient optimization capability for difficult optimization cases.

## 1. Introduction

Optimization problems are omnipresent across numerous fields within physics and engineering, and their importance cannot be overstated [1,2]. Especially in the fields of applied mechanics and engineering, finding the optimal or near-optimal solution becomes the key to enhancing the efficiency of the system, reducing the consumption of resources, and even determining the success or failure of the technology or product [3,4]. The development and application of optimization algorithms as mathematical tools play a crucial role in solving these problems [5,6]. With the rapid increase in computing power and the arrival of the significant data era, artificial intelligence technologies, such as deep learning, have developed rapidly in recent years. At the same time, they also bring new challenges and opportunities for the research and application of optimization algorithms. In deep learning, the gradient descent method and its variant algorithms, such as stochastic gradient descent (SGD), Adam, etc., have become mainstream methods in neural network training and optimization [7,8]. However, these algorithms often need to be improved to solve complex issues, such as nonconvex optimization and multimodal problems. As a class of stochastic optimization methods based on intuitive or empirical constructions, the Metaheuristic Algorithm (MHA) has shown significant advantages in solving practical optimization designs in a wide range of domains due to its lack of a strict gradient requirement, as well as its parallel search, fewer parameters, and strong search capability [9,10].

MHAs are a class of optimization tools designed for simulating natural phenomena or processes [11]. They can accurately seek optimal or near-optimal solutions in complex search spaces, an ability that is made possible by the simulation of complex behaviors, such as biological evolution, physical motions, and chemical reactions. These algorithms are capable of global search and are flexible in adapting their strategies to diverse optimization problems [12,13]. Depending on the simulation objects and mechanisms, MHAs can be categorized into four primary groups: evolution-based, physics-based, population-based, and human-based [14]. Inspired by the theory of biological evolution, evolution-based methods explore and optimize problem solution space by simulating biological evolutionary mechanisms, such as natural selection, heredity, and mutation. Among them, genetic algorithms (GA) [15] are the pioneers in this field, and they mimic the process of biological inheritance and mutation. In addition, differential evolutionary algorithms (DEs) [16], and Evolution Search (ES) [17] are well-known evolutionary algorithms. Physical phenomena and laws inspire physics-based MHAs. The Simulated Annealing Algorithm (SA) simulates the physical process of heating an object to melt and then its gradually cooling to optimize the problem solution [18]. Similarly, the Fick’s Law Algorithm (FLA) is based on Fick’s first law of diffusion [19], while the Gravitational Search Algorithm (GSA) simulates the law of gravity in the physical world [20]. On the other hand, population-based metaheuristic algorithms simulate the behavior or group intelligence of populations in nature [21]. They represent the solution of a problem as individuals in a population and find the optimal solution through interaction, competition, and cooperation among individuals. The Particle Swarm Algorithm (PSO) [22] is the earliest traditional algorithm, and it simulates the social behavior of animal groups. Other well-known algorithms include the Gray Wolf Optimization (GWO) [23], the Harris Hawk Optimization (HHO) [24], and the Black-Winged Kite Optimization Algorithm (BKA) [25]. Finally, human-based methods are mainly generated based on human behaviors, mindsets, and cognitive processes. The Political Optimization Algorithm (PO) [26], Human Evolutionary Optimization Algorithm (HEOA) [27], and Rich-Poor Optimization Algorithm (PRO) [28] are outstanding representatives in this field. These algorithms provide new ideas and methods for solving complex optimization problems by simulating human behaviors or cognitive processes.

YDSE is a method based on physical principles proposed in 2022 [29]. It has effectively addressed many realistic engineering problems due to its advantages of high flexibility, high convergence accuracy, and global search capability. Compared with some classical and new algorithms, YDSE is highly competitive and asymptotic when facing engineering optimization problems. However, YDSE cannot effectively balance the equilibrium between global and local searches, as well as having slower convergence to the optimal solution, and tends to trap in the local best, thus limiting its application scope. Some improved versions of YDSE also try to solve these problems and verify the effectiveness of the improvement through engineering optimization problems. For example, Hu et al. presented an enhanced form of YDSE. They tried to introduce four efficient mechanisms to enhance the optimization ability of YDSE and demonstrated better optimization performance in some difficult engineering optimizations [30]. A smart weighting tool for optimizing dissolved oxygen levels based on Young’s double-slit optimizer was presented by Dong et al. [31].

Although YDSE and some existing enhanced versions still demonstrate better convergence performance and results when facing engineering optimization problems, they still do not effectively balance the global and local searches and are especially prone to becoming bogged down in localized solutions when facing complex issues. This result leads to unsatisfactory optimization results obtained by the YDSE optimizer when solving difficult optimization issues. Therefore, this study proposes a fractional-order boosted hybrid YDSE optimizer to further alleviate the shortcomings of existing YDSE algorithms and attempts to solve realistic engineering optimization problems of higher complexity. In addition to the above motivations, bringing in operators and single strategies, while improving the manifestations of one aspect of the algorithm, can indirectly impair the performance of other aspects. Therefore, we jointly improve the YDSE problem by introducing multi-strategy mechanisms and minimizing this indirect damage. Second, many original and improved algorithms have been proposed that are performance-efficient and effective. However, based on the No Free Lunch (NFL) statement [32], it has been shown that existing methods are not capable of comprehensively addressing all conceivable optimization challenges. This scenario suggests that a certain category of optimization algorithms, while capable of yielding satisfactory outcomes for particular problems, may result in unacceptable results for others [33]. In addition, the fractional-order strategy is considered a strategy to efficiently address the balance between exploration and exploitation. It has been applied to improve a variety of algorithms and has demonstrated efficient experimental performance. For example, a JS algorithm based on fractional-order improvement has been proposed to address the inability of the JS algorithm to effectively balance between exploration and exploitation, and the effectiveness of the strategy has been verified through a variety of engineering optimization problems and real-world economic forecasting problems [34]. These reasons motivate this study to propose a fractional-order boosted hybrid YDSE optimizer. Solteiro Pires et al. proposed a convergence rate control method for Particle Swarm Optimization algorithms based on fractional-order calculus [35]. Luo et al. proposed a Jellyfish Search Algorithm (JSA) for the optimization and tuning of the control gains of the developed strategy to obtain a high-quality global optimum [36]. Zhang et al. proposed an improved algorithm of the Bird Flock Algorithm for solving parameter estimation of fractional-order chaotic control and synchronization [37]. These reasons motivate this study to propose a fractional-order boosted hybrid YDSE optimizer.

Therefore, this study proposes a fractional-order boosted hybrid YDSE optimizer. First, a fractional-order strategy is introduced into the dark edge position updating of FYDSE to ensure more effective exploitation of the search potential of a single neighborhood space while reducing the possibility of getting bogged down in localized solutions. Secondly, piecewise chaotic mapping is constructed in the generation of the initial solution to gain better distributed initial individuals and to enhance the speed of convergence to the most individuals. Moreover, the low exploration space of the algorithm is extended by using a dynamic inversion strategy, which improves the probability of obtaining a globally optimal solution. Finally, by introducing the vertical operator, FYDSE can better balance global exploration and local exploitation to explore new unknown areas. In addition, the presented FYDSE is compared with a series of the most efficient comparative algorithms in a comparative experiment on the CEC2022 test. The experimental outcomes and statistical analysis validate the efficient performance of FYDSE in addressing optimization issues. Further, to verify the efficient ability of the FYDSE in facing real-world engineering application problems, comparative experiments of FYDSE are conducted on three complex truss topology optimization problems. The superior adaptability and high efficiency of the FYDSE in complex engineering issues validate the ability of the FYDSE in dealing with similar applied mechanics and engineering problems.

The key contributions of this research are enumerated as follows:

(1) In this article, a fractional-order boosted hybrid Young’s double-slit experimental optimization algorithm is proposed.
(2) A multi-strategy mechanism is employed to jointly improve the YDSE problem to mitigate the negative impact of a single strategy on other aspects of the algorithm and to improve its ability to solve complex problems.
(3) Experimental results of numerical and 20-bar, 24-bar, and 72-bar topology optimization validate the performance advantages of the proposed FYDSE approach.

The remainder of the article is arranged as described below. Section 2 describes the specific details of the YDSE optimizer. Section 3 describes a fractional-order boosted hybrid improved YDSE optimization method. In Section 4, the ability of FYDSE on the CEC2022 test suit is discussed. Section 5 describes the performance of YDSE on the 20-bar, 24-bar, and 72-bar topology problems. Finally, the conclusions of this research are introduced.

## 2. The Theory of YDSE

YDSE is an innovative physics-based meta-heuristic method presented in 2022 that provides high-quality solutions to numerical and engineering optimization problems based on the fluctuating properties of light [29]. YDSE has already demonstrated excellent performance in the original and its improved versions, effectively addressing real-world mechanical optimization issues. The workflow of YDSE consists of three core steps: The first part is to initialize the position of the population. In the second part, YDSE simulates the fluctuating behavior of light according to Huygens’ principle and guides the algorithm in finding the optimal solution by constantly updating the traveling waves and path differences. The third part is the exploration and development phase. In the exploration stage, YDSE attempts to discover new potential optimal solutions by expanding the search range. Meanwhile, in the exploitation phase, the current optimal solution is fine-tuned to obtain a more highly interpretable solution. The ability to effectively balance these two processes dramatically determines the performance of the algorithm [29].

YDSE initializes each individual by simulating the process of projecting a monochromatic light wave source through two closely spaced slits into the barrier. To simulate this process, an original light source composed of *M* waves is constructed in the solution space as follows [29]:

(1) $$M_{ij}=lb_{j}+rand\times (ub_{i}-lb_{j}),\;i=1,\;2,\;\ldots ,\;n,\;j=1,\;2,\;\ldots ,\;d$$

where *M_i,j_* represents the *i*th wave in dimension *j*. In addition, *n* means the population size and *d* means the dimension of the issue. For each *j* dimension, *ub_j_* and *lb_j_* denote the maximum and minimum range of the issue. In addition, *rand* means a randomly generated value between 0 and 1.

After initialization, waves next pass through the slit barrier and diverge in many different directions. The resulting dispersion allows for constant changes in the position of the wave source and the wave center. The YDSE method does this by setting the points on the wavefronts passing through the two slits to be equal and set to *n*. In a simplified way, the wavefronts of *n* points at two slits can be calculated by the following two equations [29]:

(2) $$FS_{ij}=M_{ij}+Length\times rand_{1}\times (S_{Avg}-M_{ij})$$

(3) $$SS_{ij}=M_{ij}-Length\times rand_{2}\times (S_{Avg}-M_{ij})$$

where *FS_ij_* and *SS_ij_* denote the *j*th dimension coordinates of the *i*th point on the wavefront flowing out of the first and second slits, respectively. To simulate the random scattering phenomenon that may occur after a light wave passes through the slit, we introduce two random variables, *rand*_1_ and *rand*_2_, both of which take values in the range of [−1, 1]. *Length* means the length from the light source to the barrier. *S_Avg_* means the mean value of the current light wave group, which is calculated as follows [29]:

(4) $$S_{Avg}=\frac{1}{n}\sum_{i=1}^{n}M_{i,:}$$

where *M_i_*_,:_ means the *i*th original light source.

Points on the wavesurface propagating from the two narrow gaps produce interference patterns. Specifically, constructive and damaging interference generate light and dark streaks. Therefore, the position of each individual is updated by constructed wave fronts (*FS* and *SS*) to realize the interference behavior and path differences between light and dark streaks. The specific equations are as follows [29]:

(5) $$X_{i,:}=(\frac{FS_{i,:}+SS_{i,:}}{2})+\Delta P$$

where ∆*P* means the path length from the *FS* point to the *SS* point. In addition, *FS_i_*_,:_ and *SS_i_*_,:_ mean the *i*th *FS* point to *SS* point. The introduction of ∆*P* is utilized to distinguish different interference orders. Specifically, for zero-order or even-order interference, bright fringes are produced in the interference region. Meanwhile, for odd-order interference, dark fringes are produced. The specific equations are given below [29]:

(6) $$\Delta P=\{\begin{array}{c} 0,\;\;\;\;\;if\;bright\;and\;m=2\;\\ \frac{\lambda }{2}\cdot (2m+1),\;if\;dark\;and\;odd\;m\;\;\;\\ n\lambda ,\;\;\;\;if\;bright\;and\;even\;m \end{array}$$

where *m* means the order of interference. In addition, *λ* denotes wavelength.

The bright fringe emerges due to the combination of two waves, creating a wave of greater amplitude. The amplitude of this bright fringe is then progressively updated as follows [29]:

(7) $$A_{b}(t)=\frac{2}{1+\sqrt{1-{\left(t\times \cosh(\pi /t)/T_{\max}\right)}^{2}}}$$

where *A_b_* means the mean amplitude of bright fringes. In addition, cosh(·) means the cosh function. *t* represents the now iteration and *T*_max_ means the maximum iterations.

If the peak of one wave meets the trough of the other when two waves are added together, they both cancel out and produce dark fringes. Similarly, the amplitude of the dark fringe is iteratively updated as follows [29]:

(8) $$A_{d}(t)=\phi \times \tanh^{-1}(-\frac{t}{T_{\max}}+1)$$

where *φ* represents a constant with a value of 0.38. In addition, tanh^−1^(·) means the inverse of the tanh function.

Both bright and dark fringes represent potential candidate solutions when exploring solutions. In particular, the center fringe location is considered to be the optimal solution. Due to the effect of destructive interference, the fitness values of the dark fringe candidate solutions are lower than those of the bright fringe candidate solutions. This interference causes the positions in the dark fringe to be inferior to objective function than those in the light fringe. Therefore, YDSE emphasizes the priority of exploring for hopeful solutions in the dark zone during the exploration phase. A dark edge update is introduced to guide the search process and ensure that the solution space can be explored more efficiently. The specific update equation is as follows [29]:

(9) $$X_{N-odd}(t+1)=X_{N-odd}(t)-(rand\times A_{d}(t+1)\times Li_{N-odd}(t+1)\times X_{N-odd}(t)-Z\times X_{best}(t))$$

where *X_N-odd_* means the dark fringe, and *rand* denotes a randomly selected number between 0 and 1. *Li_N-odd_* denotes the dark edge intensity, which is utilized to reflect the brightness change of the dark fringe and *X_best_* denotes the best solution. In addition, *Z* means a trial vector of *d*, as follows [29]:

(10) $$Z=\frac{t^{2\times rand-1}}{h}$$

where *h* means a randomly created number in the range of [−1, 1]. In addition, the definition of *Li_N-odd_* is as follows [29]:

(11) $$Li_{N-odd}(t+1)=Li_{\max}(t+1)\times \cos^{2}(\frac{\pi d}{\lambda L}y_{d}(t+1))$$

where *y_d_* is employed to measure the length from the center stripe to the dark stripe, as follows [29]:

(12) $$y_{d}=\frac{\lambda L}{d}(m+\frac{1}{2})$$

In addition, *Li_max_* denotes the maximum center stripe intensity, as follows [29]:

(13) $$Li_{\max}(t+1)=C\times \frac{t}{T_{\max}}$$

where *C* is taken as 10^−20^, which represents the maximum intensity.

When the order is even, YDSE pays special attention to the bright edge regions and finds promising candidate solutions in them. It assumes that these regions are where the best solutions are potentially located, which, in turn, generates constructive interference in favor of the solution process. Therefore, during the development phase, the YDSE optimizer spares no effort in exploring all possible and promising candidate solution regions in the bright edge region. Its position update equation is concretely given as below [29]:

(14) $$X_{N-even}(t+1)=X_{N-even}(t)-((1-g)\times A_{b}(t+1)\times Li_{N-even}(t+1)\times X_{N-even}(t)+g\times Y)$$

where X*_N-__even_* means the bright fringe. In addition, *Y* denotes the difference between two randomly acquired stripes and *Li_N-__even_* denotes the intensity of the bright stripe. *g* means a randomly created number in the range of [0, 1]. The definition of *Y* is as follows [29]:

(15) $$Y=X_{N-r1}(t)-X_{N-r2}(t)$$

where X*_N−r1_* and X*_N−r2_* represent two random fringes. In addition, the definition of *Li_N-even_* is as follows [29]:

(16) $$Li_{N-even}(t+1)=Li_{\max}(t+1)\times \cos^{2}(\frac{\pi d}{\lambda L}y_{b}(t+1))$$

where *Li_max_* is defined through Equation (13).

Finally, the specific formula for the position update method of the center fringe is as follows [29]:

(17) $$X_{N-zero}(t+1)=X_{best}(t)+(A_{b}(t+1)\times Li_{\max}(t+1)\times X_{N-zero}(t)-rand\times Z\times X_{rb}(t))$$

where *X_N-zero_* means the central fringe and *rand* means a random number between 0 and 1. In addition, X*_rb_* represents the bright fringe selected based on a random even number of *rb*.

## 3. Proposed FYDSE Algorithm

The YDSE method suffers from slow convergence, is easily caught up in localized solutions, and is unable to effectively balance exploration and exploitation when facing complex optimization problems in engineering. In this work, a fractional-order improved optimization method for a multi-strategy YDSE is proposed. The specific modifications of the proposed FYDSE are shown below.

### 3.1. Fractional-Order Modified Mechanism

Fractional calculus elevates the fundamental order of calculus by extending its realm from integers to fractions. It systematically derives the solution limit by employing differential approximations of integer calculus, specifically through the differentiation and integration of fractional orders [34,35]. The introduction of the fractional-order strategy in the population position updating process of FYDSE, which extends *X*(*t*) to fractions through the fractional-order formula, guarantees a more efficient exploration of the potential within a single neighborhood space, thereby minimizing the likelihood of falling into a local optimum. The definition of fractional-order discretization based on the Grunwald–Letnikov (G-L) method [38] is summarized as follows [34]:

(18) $$D^{\beta }[\omega (t)]=\frac{1}{T^{\beta }}\sum_{k=0}^{r}\frac{{\left(-1\right)}^{k}\Gamma (\beta +1)\omega (t-kT)}{\Gamma (k+1)\Gamma (\beta -k+1)}$$

where *β* is the fractional element in the common signal *ω*(*t*), Γ denotes the gamma function, and *T* is the truncation term. *r* stands for the order.

The results of the fractional derivative are closely related to the value of the current term and the previous state, with the effect of past events diminishing over time. Then, the above equation is modified as follows [34]:

(19) $$\begin{array}{l} D^{\beta }[\omega (t+1)]\approx \omega (t+1)-\beta \omega (t)+\frac{1}{2}\beta (\beta -1)\omega (t-1)-\frac{1}{6}\beta (\beta -1)(\beta -2)\omega (t-2)\\ \;\;\;\;\;\;\;+\frac{1}{24}\beta (\beta -1)(\beta -2)(\beta -3)\omega (t-3) \end{array}$$

where *β* is set to 4 [34] in the specific implementation.

**Figure 1:**
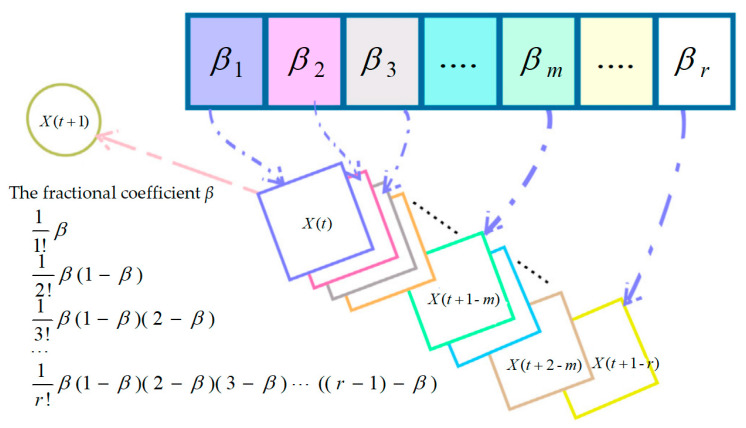
Schematic of the fractional-order modification strategy.

During the exploration phase, given YDSE’s emphasis on identifying promising solutions in the dark region, a fractional-order correction strategy was implemented to enhance the updating formula for dark streaks, thus refining our search approach. Figure 1 shows the effect of fractional-order correction on the update. The dark fringe update formula based on fractional-order correction is as follows:

(20) $$\begin{array}{l} X_{N-odd}(t+1)=\beta X_{N-odd}(t)-\frac{1}{2}\beta (\beta -1)X_{N-odd}(t-1)-\frac{1}{6}\beta (\beta -1)(\beta -2)X_{N-odd}(t-2)\\ +\frac{1}{24}\beta (\beta -1)(\beta -2)(\beta -3)X_{N-odd}(t-3)-(rand\times A_{d}(t+1)\times Li_{N-odd}(t+1)\times X_{N-odd}(t)-Z\times X_{best}(t)) \end{array}$$

Notably, upon multiplying the terms of 1/120, 1/720, or greater by the remaining components of the equation, their respective results become insignificant, scarcely influencing location updates. Consequently, these higher-order terms are disregarded.

### 3.2. Piecewise Chaotic Map Strategy

YDSE guides the updating of candidate solutions by iterative a priori information. Therefore, a better initial value captures valid information faster and thus searches for the optimal solution location faster [39]. The initialization of the established methods is through Gaussian distribution in order to generate randomly. However, this initialization is highly contingent, and there are more invalid solutions, thus limiting the effective updating of the solution. To obtain a better distribution of the initial solution space, FYDSE introduces a piecewise chaotic mapping distribution to initialize the solution space [40]. The population initialization in the piecewise chaotic mapping is achieved through chaotic variables rather than relying on random variables, thereby facilitating a full and efficient exploration of the solutions space [41]. The updated formula for the piecewise chaotic mapping is as follows [41]:

(21) $$\pi (k+1,d)=\{\begin{array}{c} \frac{\pi (k)}{d},\;\;\;\pi (k)\in [0,d)\;\;\;\\ \frac{\pi (k)-d}{0.5-d},\;\;\pi (k)\in [d,0.5)\;\;\\ \frac{1-\pi (k)-d}{0.5-d},\;\pi (k)\in [0.5,1-d)\;\\ \frac{1-\pi (k)}{d},\;\;\pi (k)\in [1-d,1)\;\; \end{array}\;\pi (k)\in [0,\;1]$$

where *d* means a random value in the scope of (0, 0.5). π(k+1,d) means the piecewise chaotic mapping function. Therefore, the initialization method is updated as follows:

(22) $$M_{ij}=lb_{j}+\pi (k,d)\times (ub_{i}-lb_{j}),\;i=1,\;2,\;\ldots ,\;n,\;j=1,\;2,\;\ldots ,\;d$$

### 3.3. Dynamic Opposition (DO)

When solving complex optimization problems, YDSE often fails to efficiently traverse the search region of low exploration, which results in the algorithm failing to capture the global optimal solution. Therefore, a dynamic opposition (DO) strategy is introduced in the YDSE optimizer [42]. It extends the search space of low exploration by using a dynamic inversion strategy, thereby enhancing the likelihood of getting close to the global solution.

Considering that frequent DO strategy updates can affect the development performance of the algorithm, a jump rate (*Jr*) determines DO policy in the current iteration or not. If the number created at random is less than *Jr*, the DO implementation strategy is applied to all individuals. Consequently, *Jr* governs the likelihood of executing DO [43]. This approach, the Randomized Jump Strategy (*RJS*), facilitates the balanced exploration and development of methods in an effective manner through iterative selection. Furthermore, implementing DO during the selection iteration leads to an enhanced utilization of the search space, thus maximizing its efficiency. The DO strategy first requires generating the opposite solution in the neighborhood of the candidate solution *X* as below [42]:

(23) $${\bar{X}}_{i,j}=lb_{j}+ub_{j}-X_{i,j},\;j=1,\;2,\;\ldots ,\;d$$

where ${\bar{X}}_{i,j}$ represents the inverse solution.

The position of the candidate solution undergoes a shift due to the influence of a randomly generated number within the range of [0, 1], thereby yielding a new position designated as *X_r__and_* [42]:

(24) $$X_{rand}=rand(\bar{X}),\;rand\in [0,\;1]$$

where *rand* means a random number.

When the candidate solution *X* is different from the moving direction of *X_r__and_* guided by the random number, a new candidate position based on the DO policy is constructed, as follows [43]:

(25) $${\bar{X}}_{do}=X+rand(X_{rand}-X)$$

where ${\bar{X}}_{do}$ represents the new position after dynamic opposition update.

To further illustrate the specific implementation of the DO strategy, Algorithm 1 provides pseudo-code for the DO strategy.
**Algorithm 1:** Dynamic Opposition Strategy1: **input**: Jumping rate *J_r_*, solution size *N*, candidate solution location *X*.2:    **if**
*rand* < *Jr*3:        **for**
*i* = 1 to *N* **do**4:              ${\bar{X}}_{i,j}=lb_{j}+ub_{j}-X_{i,j},\;j=1,\;2,\;\ldots ,\;d$5:              $X_{rand}=rand(\bar{X}),\;rand\in [0,\;1]$6:              ${\bar{X}}_{do}=X+rand(X_{r\mathrm{and}}-X)$7:          **end for**8:    **end if**9: **output:** New location on dynamic dichotomy.

### 3.4. Vertical Crossover Operator

To address the tendency of the YDSE algorithm to converge prematurely into local optimal solutions while tackling intricate problems, we develop an update strategy based on the vertical operation mechanism [44,45]. Specifically, the vertical operator performs an arithmetic crossover operation between two dimensions of the candidate solutions to generate new candidate individuals. By integrating the vertical operator, the FYDSE method gains enhanced global exploration and local exploitation abilities, allowing it to delve into novel and unexplored domains [46]. Furthermore, the adoption of the updated foraging strategy mitigates the issue of stagnant updates across iterations over many times. Assuming that the vertical crossover operation is performed with the *d*_1_ and *d*_2_ dimensions of the *i*th candidate solution, the generated conditioning solution $V_{id_{1}}^{vc}$ is as follows [45]:

(26) $$V_{id_{1}}^{vc}=c\cdot X_{id_{1}}+(1-c)\cdot X_{id_{2}},\;i\in M(1,n),\;d_{1},d_{2}\in M(1,d),$$

where *c* means uniformly distributed random values ranging from 0 to 1, *n* means the quantity of agents, and *d* signifies the dimensionality of the population. In addition, X*_id_*_1_ and X*_id_*_2_ denote the *d*1st and *d*2nd dimensions of the *i*th position. *M*(1, *n*) represents a function that selects individuals from all populations.

The vertical operator strategy is normalized for individuals, considering the upper and lower bounds for decision variables. The vertical operation of each is tailored specifically to a single individual, ensuring a focused search. This single-individual approach prevents the disruption of potentially globally optimal dimensions by abruptly departing from a locally optimal yet stagnant dimension. Furthermore, introducing a competition operator fosters healthy competition between the new and original candidate solutions. Specifically, the competition operator works as follows [46]:

(27) $$X_{\mathrm{new}}=\{\begin{array}{c} V^{vc},if\;f(V^{vc})<f(X),\\ X,\;if\;f(V^{vc})>f(X), \end{array}$$

where *V^vc^* represents the candidate solution after the vertical operator operation. *X_new_* means new position. *f*(·) represents the fitness function.

### 3.5. The Proposed Enhanced FYDSE Meticulous Steps

Given the vast search space encountered in engineering optimization problems, attaining global convergence for intricate optimization challenges poses a significant challenge. To address this, this paper introduces a fractional-order improved boosted hybrid YDSE, explicitly tailored for solving complex optimization issues. First, a fractional-order strategy is introduced into the dark fringe position updating of FYDSE to ensure a more elegant search potential in a single neighborhood space, and also to minimize the risk of drifting into local optimality. Second, during the initialization phase, a piecewise chaotic mapping is introduced to yield a superior-quality initial population, thereby enhancing the convergence speed of the algorithm. Moreover, the lower exploration space for algorithms is extended by using a dynamic inversion strategy, which improves the probability of obtaining a globally optimal solution. Finally, by introducing the vertical operator, FYDSE can enhance equilibrium global exploration and local exploitation and explore new unknown areas.

The primary processes of FYDSE are described below:

Step 1: Initialization of the relevant elements of the FYDSE: the stripe quantity *n*, the dimension *d*, the lower and upper limits of the variable *lb* and *ub*, the maximum iteration number *T*_max_, the fraction coefficient *β* and the jump rate *Jr*;

Step 2: Initialize wavefronts by piecewise chaotic mapping. Obtain the position of each fringe based on the wavefronts and path difference ∆*L* and compute the objective function value.

Step 3: Update *Li_max_* by utilizing Equation (13) and pick the optimal location *X_best_*.

Step 4: When *m* is 0, the central stripe amplitude is updated using Equation (7) and the central stripe position is also updated according to the amplitude and the best position Xbest by Equation (17).

Step 5: When *m* is *odd*, the dark streak intensity and amplitude are updated using Equations (7) and (11). Afterwards, a new location is obtained by the dark streak update formula based on the fractional-order correction.

Step 6: When *m* is *even*, Equations (8) and (16) are utilized to update the amplitude and intensity of the bright streaks and further update the location of the bright streaks.

Step 7: Examine the lower and upper bounds of the variables and evaluate the objective function of the updated solution.

Step 8: If *rand* < *Jr*, update the dynamic opposite solution ${\bar{X}}_{do}$ for all the stripes by Equations (23)–(25) and compute the fitness value.

Step 9: A vertical operator is utilized to intersect the two dimensions of a randomly selected location and the availability of that new candidate solution is determined by a competitive operator.

Step 10: Consider the limitations of the assessment variables, both the upper and lower boundaries, and evaluate the solution of the objective function accordingly. Output the optimal fringe position *X_best_*.

**Figure 2:**
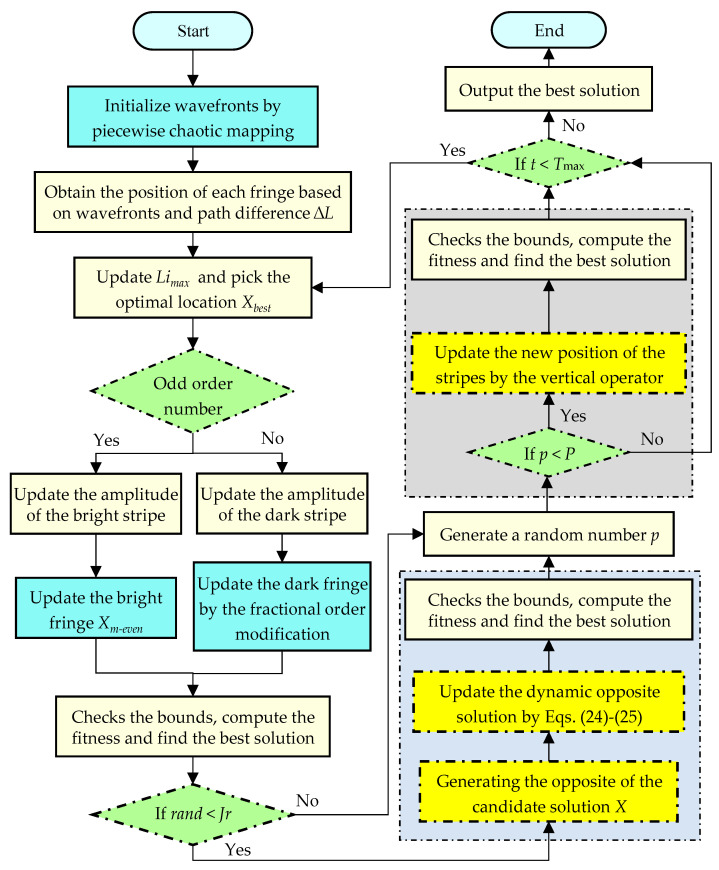
Main flowchart of FYDSE.

The figure depicted in Figure 2 outlines the proposed FYDSE flowchart; furthermore, Algorithm 2 provides the corresponding pseudo-code for a clearer understanding of its implementation.
**Algorithm 2:** Fractional-order boosted hybrid Young’s double-slit experiment optimizer1: **Input:** Initialize the related parameters of the proposed FYDSE algorithm: number of fringes (*n*), upper and lower bounds (*lb* and *ub*), dimension (*d*), maximum number of iterations (*T*_max_), the fraction coefficient *β* and jump rate *Jr*;2: Initialize a monochromatic light source consisting of *n* waves using Equation (22) by piecewise chaotic mapping.3: Calculate the wavefronts (*FS* and *SS*) of the two slits.4: Obtain the position of each fringe based on wavefronts and path difference ∆*L*.5: Examine the upper and lower bounds of the variables and evaluate the objective function of the updated solution.6: **While** t *< T*_Max_ **do**7:    Update *Li_max_* by utilizing Equation (13) and pick the optimal location *X_best_*8:    **For** *i* =1 to *n* **do**9:          Update *Z* with Equation (10).10:          **If** *m* = 0 (Central fringe)11:              The intensity and amplitude of the central fringe are updated with Equations (17) and (7).12:              Update the position of the center stripe *X_m_*_-*zero*_ based on the amplitude and the optimal position *X_best_*.13:          **Else if**
*m* = *even* (bright fringe)14:              The intensity and amplitude of the bright fringe are updated with Equations (14) and (7).15:              Update the bright fringe *X_m_*_-*even*_ by Equation (14).16:          **Else if**
*m* = *odd* (dark fringe)17:              The intensity and amplitude of the dark fringe are updated with Equations (9) and (8).18:              Update the new position *X_m_*_-*odd*_ of dark fringe based on the fractional-order modification with Equation (20).19:**          End if**20:          Check the bounds of the variables.21:    **End for**22:    **If**
*rand* < *Jr*23:        **For**
*i* = 1 to *n* **do**24:                ${\bar{X}}_{i,j}=lb_{j}+ub_{j}-X_{i,j},\;j=1,\;2,\;\ldots ,\;d$25:                $X_{rand}=rand(\bar{X}),\;rand\in [0,\;1]$26:                ${\bar{X}}_{do}=X+rand(X_{rand}-X)$27:          **End for**28:    **End if**29:    B = permutate(d).30:    **For**
*i* = 1 to *d*/2 **do**31:        Update a uniformly random value *p*.32:        **If**
*p*< *P* then let *no*1 = *B*(2*i*−1), and *no*2 = *B*(2*i*).33:            **For**
*j* = 1 to *n* **do**34:                  Construct a random value *c* in the range of [0, 1].35:                  Update the position of a stripe according to the vertical operator.36:            **End for**37:        **End if**38:    **End for**31:    Update the best position *X_best_*.32:    *t = t +* 1.33: **End while**34: **Output:** the best solution *X_best_*.

### 3.6. The Computational Complexity of FYDSE

An important metric for evaluating optimization algorithms is the time complexity. The original YDSE has a complexity of *O*(*T*_max_×*M*×*n*+*T*_max_×*n*×*d*), where *M* represents the number of evaluations of the objective function. The proposed FYDSE includes four effective strategies. First, the segmented mapping initialization has the same complexity of *O*(*n*×*d*) as the Gaussian-based initialization of YDSE. Second, the fractional-order improved dark streak updating strategy has no added time complexity. Since the fractional-order improved strategy needs to save the experimental results of the first *β* iterations, the space complexity increases by *O*(*β*×*n*×*d*). Second, the DO strategy produces dynamic inverse solutions for all candidate solutions. However, given the limitation imposed by *rand* < *Jr*, the computational complexity of the DO strategy in the crap scenario of fulfilling the equation stands at *O*(*T*_max_×*n*×*d*). Finally, the complexity of the vertical operator is *O*(*T*_max_×*n*/2×*d*). Thus, the overall complexity of FYDSE is:

(28) $$\begin{array}{l} O(FYDSE)=O(T_{\max}\times M\times n+T_{\max}\times n\times d)+O(n\times d)+O(\beta \times n\times d)\\ \;\;\;\;\;\;+O(T_{\max}\times n\times d)+O(T_{\max}\times n/2\times d)\\ \;\;\;\;\;\;=O(T_{\max}\times M\times n+T_{\max}\times 5n/2\times d) \end{array}$$

## 4. Numerical Simulations

To comprehensively measure the capabilities of FYDSE, by contrast to some other SOTA methods, in this section a comparative experiment on the CEC2022 set of tests is described. All the methods are set with the same parameters in the experiments, i.e., the maximum iteration number is 1000, and the population size is 30. We chose the mean, worst solution, optimal solution, and standard deviation as the evaluation indexes to visualize the experimental results. The experimental results are presented and analyzed in various forms, such as iterative plots, box plots, and radar plots. All tests were conducted on a personal computer with Matlab-2019b with a 2.11 GHz quad-core Intel(R) Core(TM) i5 and 8.00 GB.

### 4.1. Experimental Parameter Setting and Benchmark Functions

To deeply verify the effectiveness and superiority of our proposed FYDSE algorithm, we identified and selected a set of advanced and distinctive optimization algorithms for a comprehensive analysis and comparison with the experimental findings of FYDSE as follows: (1) YDSE [29]; (2) Artificial Hummingbird Algorithm (AHA) [47]; (3) Snake Optimization (SO) [48]; (4) Dandelion Optimizer (DO) [49]; (5) Aquila Optimizer (AO) [50]; (6) Chimpanzee Optimization Algorithm (ChOA) [51]; (7) Honey Badger Algorithm (HBA) [52]; (8) Sand Cat Swarm Optimization (SCSO) [53]; (9) Salp Swarm Algorithm (SSA) [54]; (10) White Shark Optimizer (WSO) [55]; (11) COOT Algorithm (COOT) [56]. By comparing with these algorithms, we aim to comprehensively demonstrate the advantages and features of the FYDSE algorithm in terms of performance, efficiency, and adaptability, thus further proving its capability to solve sophisticated optimization issues. Table 1 provides the parameters for the various algorithms.

The CEC2022 test suite contains 12 well-designed single-objective test functions used as benchmark functions for this experiment. These functions cover a wide range of types, such as single-peak, basis, hybrid, and combined functions, which are distinctive in complexity and morphology. Some functions exhibit smooth surface features, while others are full of sharp peaks and steep valleys. Among them, for the cec01 function, as a representation of a single-peaked function, only one globally optimal solution can measure the convergence speed and accuracy within the algorithm. On the other hand, the cec02-cec05 functions represent multimodal functions that possess numerous local optima, among which a single global optimum is designed to test whether the algorithm can successfully avoid traps of local minima and thus find the globally optimal solution. In addition, the hybrid functions cec06-cec08 are used to model the properties of real-world complex problems and allow for the evaluation of the algorithm’s effectiveness and ability to solve problems with hybrid properties. The CEC2022 test suite also introduces several combinatorial functions, such as the cec09-cec12 functions, which incorporate characteristics from a diverse set of optimization problems, presenting more intricate choices for the algorithms to overcome. In addition, the suite contains several constrained optimization problems. These problems search for optimal solutions under specific constraints and require the algorithms to adhere to these constraints strictly during optimization. The value domains of all test functions are set between [−100, 100].

### 4.2. Analysis of Exploration and Exploitation Behaviors

In the conventional YDSE framework, the refinement of dark fringes emphasized probing unknown areas to foster diversity. In contrast, the center and bright fringe prioritize cultivating optimal solutions within promising intervals. On the other hand, FYDSE improves the dark streak updating process through a fractional-order correction strategy, so that the positional updating of dark fringes focuses more on the search potential of a single neighborhood space, which mitigates the risk of converging to a local optimum and aids in discovering an optimal solution. Meanwhile, performing dynamic opposition for all fringe positions facilitates the exploration of the inverse positions of candidate solutions in the domain of the search, increasing the possibilities for searching undeveloped areas. Furthermore, incorporating the vertical operator enhances the exploration of potentially rewarding regions by augmenting the exploration of stripe positions within the dimensional intersection.

To ascertain the efficacy of the implemented strategy in balancing the exploration-exploitation equilibrium, we delve into the group diversity of FYDSE in the context of CEC2022. Population diversity infers group traits within a condensed environment through variations among individual dimensions [57]. Specifically, an increase in the difference between dimensions of the stripe location determines the dispersal or distribution in the search area of the population. Conversely, individuals will cluster into a convergence region. The introduction of the vertical operator improves the difference between the dimensions of the stripe positions.

First, we define the diversity (*Div_j_*) of the *j*th dimension and further compute the average diversity (*Div*) of the dimensions. Further, the proportion of exploration and exploitation is calculated based on the average diversity. The specific formula is as follows [57]:

(29) $$Div_{j}=\frac{1}{n}\sum_{i=1}^{n}Median(X_{:,j})-X_{i,j}$$

(30) $$Div=\frac{1}{d}\sum_{j=1}^{d}{Div}_{j}$$

where *n* represents the number of all light and dark stripes and *d* represents the dimension. The median of the *j*th dimension across all (*n*) stripes is denoted as *Median*(*X*_:,*j*_).

Meanwhile, the definitions of the exploration rate *Explora*(%) and the exploitation rate *Exploita*(%) are given [57]:

(31) $$Explora\;(\%)=\frac{Div}{\max(Div)}\times 100$$

(32) $$Exploita\;(\%)=\frac{\left|Div-\max(Div)\right|}{\max(Div)}\times 100$$

where max(·) denotes the max function.

**Figure 3:**
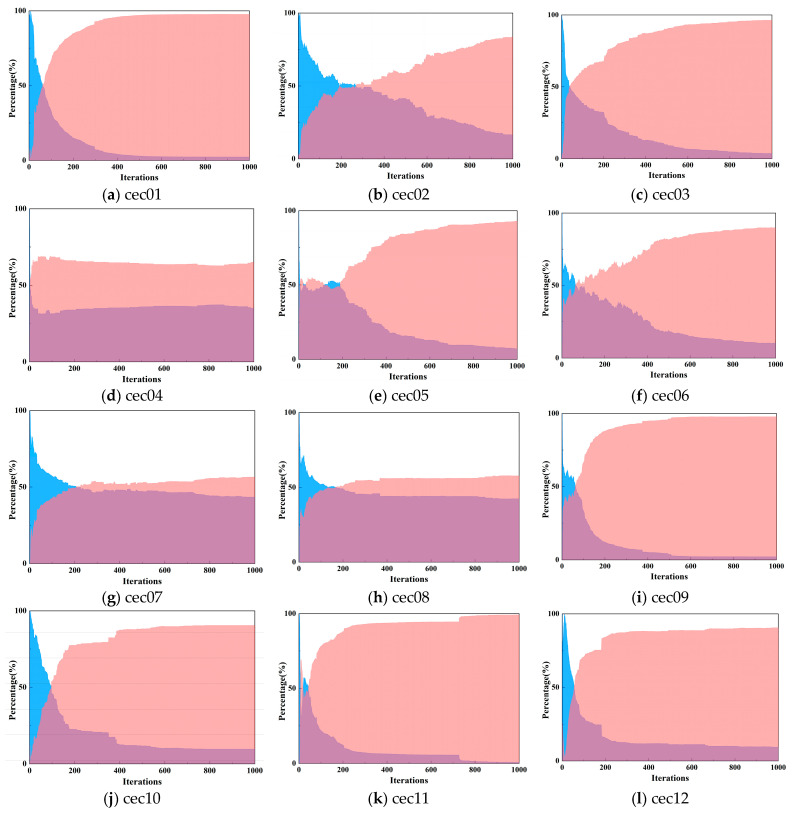
Iterative plots of exploration and exploitation rates of the FYDSE algorithm for 12 test functions in CEC2022.

This experiment validates the diversity results of FYDSE at the CEC2022 test function with 1000 iterations of FYDSE. Figure 3 shows the iterative plots of the exploration and exploitation rates of FYDSE at CEC2022 for all 12 test functions. From the analysis of the graphs, it can be concluded that for cec02, cec05, and cec07, FYDSE exhibits a high rate of exploration during extended iterations, while maintaining a high utilization rate during the middle and later stages of iterations. This result is attributed to the fact that the piecewise mapping initialization and dynamic opposition strategies enhance the search potential of YDSE. Meanwhile, the fractional-order modification strategy enhances the exploration performance by improving the update of dark streaks and effectively utilizing the existing prior knowledge. When dealing with cec01, cec03, cec09, and cec11, most iterations tend to be in a more intensive exploitation phase, with only a brief exploration phase in the initial stages. This is primarily due to the vertical operator enhancement of the exploitation stage. Additionally, the lower exploration rate during shorter iteration times indicates that the modified strategy aids the algorithm in swiftly locating the best solution.

### 4.3. Results Analysis with the Latest Methods in CEC2022

Table 2 provides the experimental outcomes of the FYDSE with the other latest comparison algorithms on the CEC2022 test dataset. To comprehensively evaluate the performance of these algorithms, we have chosen five key evaluation metrics, including the mean, the best value, the worst value, the standard deviation, and the ranking, to consider these algorithms comprehensively. These metrics provide us with a multi-dimensional view of the algorithm performance, which helps us to understand more clearly the performance differences and advantages and disadvantages between the FYDSE algorithm and other algorithms. As is evident from Table 2, the FYDSE algorithm exhibits the superior average performance among the eight test functions (cec02, cec06, cec07, cec08, cec09, cec10, cec11, cec12) under consideration, significantly outperforming the other algorithms. Not only that, but FYDSE also demonstrates remarkable competitiveness in other test functions. Comparatively, the original algorithm YDSE provides the best average results only on the specific test function cec05. The HBA algorithm, on the other hand, achieves the best average performance on two test functions, cec01 and cec03. In addition, the WSO algorithm gives the best average results for the cec04 test function. And for the cec01 function, both the HBA and SSA algorithms find the optimal solution. When exploring the cec01 single-peak function, both the HBA and SSA algorithms can converge to the global optimal solution. Meanwhile, our proposed FYDSE algorithm can also reach the sub-optimal solution level, further verifying that the FYDSE algorithm is not only fast-converging but also highly accurate. When confronted with these multimodal functions from cec02 to cec05, FYDSE achieves the best mean result in the cec02 function. Although the best mean value is not obtained on several other functions, the test results of FYDSE show the most minor standard deviation, demonstrating the stable exploration and development capability of FYDSE when facing multimodal functions. It is especially worth mentioning that the FYDSE algorithm can also find the global optimal solution when solving combinatorial and hybrid function problems. This achievement highlights the global solid search capability of FYDSE when solving complex, multivariate optimization problems and demonstrates its capability to achieve a balance in exploration and exploitation. In particular, when handling higher-dimensional, multimodal optimization tasks, the outstanding performance of FYDSE allows it to address the intricacies of real-world optimization problems with more excellent proficiency.

Table 3 provides a comprehensive overview of the statistical outcomes obtained from the Wilcoxon rank sum test conducted on FYDSE and alternative algorithms for comparison, with a predefined significance level of 0.05. Here, we describe the performance difference between the algorithms based on the size of the *p*-value. Specifically, when the *p*-value is less than 0.05, the difference between FYDSE and the comparison algorithm is significant. In contrast, when the *p*-value is greater than or equal to 0.05, it implies that the difference in performance between the two is insignificant. In addition, the symbols “-”, “=”, and “+” in Table 3 provide us with intuitive performance comparison information. Among them, “-” indicates that other meta-heuristic algorithms are not as practical as FYDSE in the corresponding test function. “=” indicates that FYDSE and the comparison algorithm have comparable performance in this test function, and both of them have the same effect. “+” indicates that the other algorithms obtained better results than FYDSE.

Upon examining Table 3, we can discern the distribution of the outcomes for all algorithms in the Wilcoxon test. Specifically, the results of the AHA, SO, HBA, and SSA algorithms are each 0/1/11. The statistical results imply that FYDSE does not have statistically worse results than AHA and SO in this set of tests and demonstrates better yield outcomes in a majority of the test functions. On the other hand, the results of the DO, SCSO, AO, and ChOA algorithms are 0/0/12. The statistical results imply that these algorithms neither show better performance than FYDSE nor comparable performance to FYDSE in the tests, but instead show worse performance 12 times. In addition, the Wilcoxon test results for both WSO and COOT are 0/2/10, indicating that they outperform the proposed FYDSE algorithm for no test function in the statistical test, and that their performance is comparable for the two test functions. Finally, from the statistical results of the original YDSE, it can be found that only one test function exists for YDSE that is statistically superior to FYDSE. However, in the other test function, FYDSE obtains superior experimental results. These detailed test results illustrate the performance differences between the algorithms.

**Figure 4:**
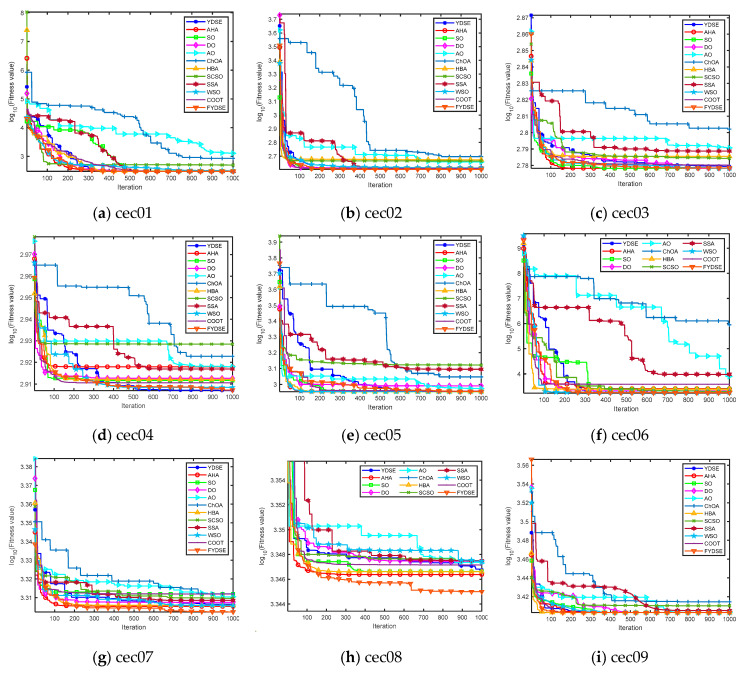

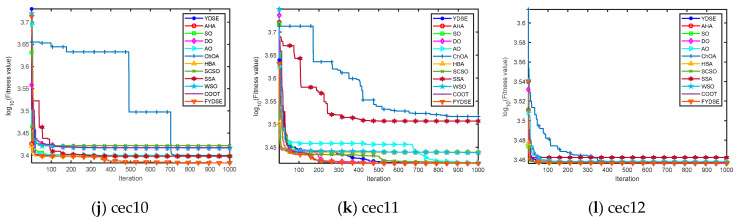
Plot of convergence of FYDSE and another comparative algorithm on the CEC2022 benchmark function.

Figure 4 showcases the convergence performance of the FYDSE in comparison to other approaches when evaluated on the cec2022 test function set. The x-axis represents the number of iterations, whereas the y-axis represents the fitness values, where some function results are expressed in logarithmic form with a base of ten. All algorithms start from the same initial point (i.e., iteration zero) to ensure a fair comparison. Observing Figure 4, for the cec01-cec03 functions, FYDSE exhibits a high convergence rate during the initial iteration phase, followed by a gradual localization of the optimal position and validation of the previous findings by updating the position. For the cec04-cec06 functions, FYDSE subsequently obtains the best solution at a satisfactory speed and executes a precise search optimization in proximity to the optimal solution. This performance proves its reliability in avoiding local optima. For the cec07-cec08 function, FYDSE can steer the search over a large area during the initial iteration phase to find potentially high-quality regions in the search space. FYDSE shifts to a localized search as the algorithm proceeds, updating to the optimal location over a smaller area. For the cec09-cec12 functions, FYDSE can swiftly transition between the initial search phase and later stages of development, converging to a solution that is close to optimal at an early iteration. Afterward, it continues pinpointing the optimal position and validating previous observations by updating the results. In summary, FYDSE performs well in all four types of test functions and maintains a significant advantage in most of them. Moreover, these compelling results also demonstrate that the FYDSE algorithm effectively strikes a harmonious balance between exploratory and developmental search strategies.

**Figure 5:**
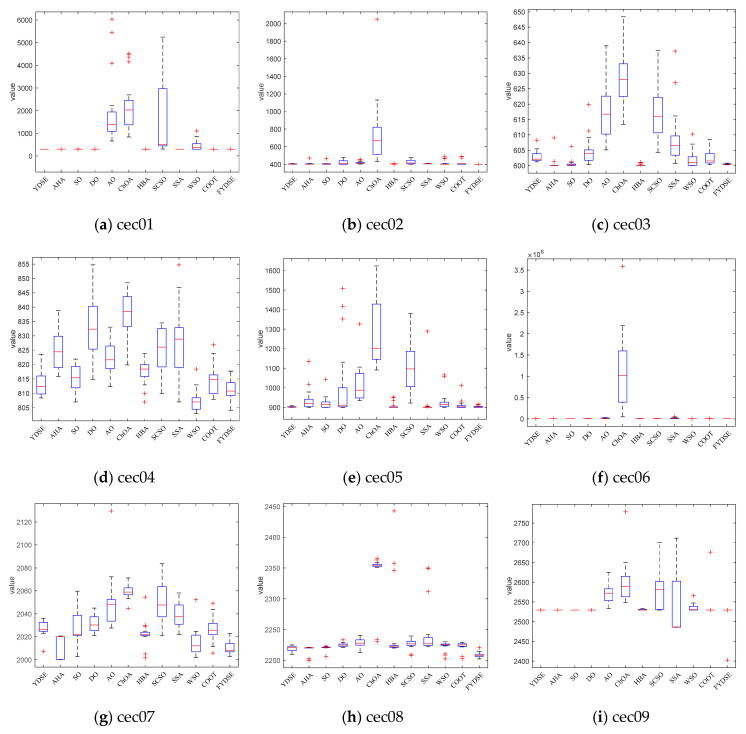

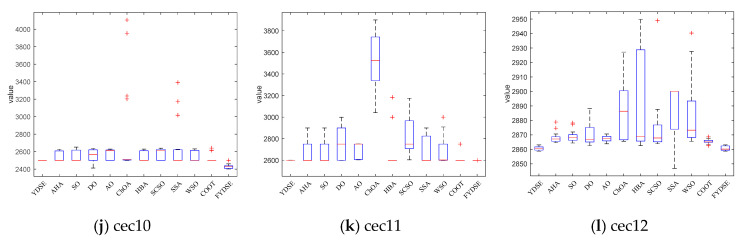
Box plot of FYDSE versus another comparative algorithm on the CEC2022 test function set.

Figure 5 shows the distribution of the best mean values of FYDSE and other comparison algorithms in multiple test functions through box-and-line diagrams. From the figure, we can see that in most test scenarios, the distribution of the best mean values of FYDSE is more concentrated and compact, fully demonstrating the excellent performance of FYDSE. In addition, this distribution characteristic further proves that FYDSE has excellent consistency and stability under different testing conditions, making it stand out amongst the algorithms. Specifically, when facing the test functions of cec03, cec04, cec07, cec08, and cec12, the box-and-line plot box of FYDSE is narrower, which implies that the performance of FYDSE fluctuates less during multiple iterations, demonstrating its stability and reliability. As for the test functions cec01, cec02, cec05, cec09, cec10, and cec11, the box-and-line diagram of FYDSE shows a red line, which means that FYDSE can solve the problem effectively and achieve a high-performance level during each iteration. This performance once again proves the superiority and usefulness of FYDSE.

**Figure 6:**
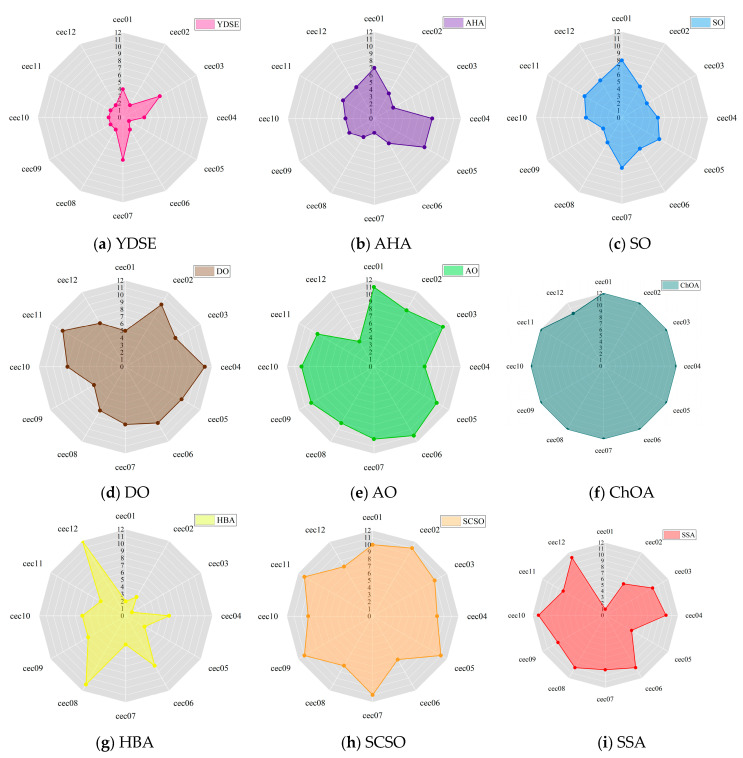

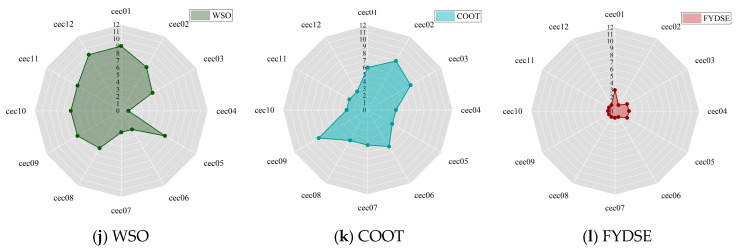
Bar plot of FYDSE versus another comparative algorithm on the CEC2022 test function set.

Figure 6 visually depicts the capability rankings of the FYDSE against a string of comparative algorithms across the twelve functions of the CEC2022 benchmark test set, utilizing an intuitive radar chart format. Upon examining Figure 6, it becomes evident that the FYDSE exhibits a significantly smaller coverage area within the radar chart, which not only accentuates its outstanding performance across various optimization scenarios but also attests to its exceptional optimization capabilities and stability. Regardless of whether it tackles simple or complex optimization problems, FYDSE can swiftly discover satisfactory solutions through efficient and accurate search strategies.

### 4.4. Comparison of Algorithm Complexity and Running Results

In order to validate the proposed FYDSE algorithm against other algorithms in terms of complexity and running results, we provide the algorithmic complexity of the proposed algorithm and other methods in Table 4, where *T*_max_ represents the maximum iteration, *M* represents the number of function evaluations, *n* represents the number of populations, and *d* represents the dimension.

The table shows that the YDSE, SO, HBA, SSA, WSO, and COOT algorithms have the same complexity. Also, the DO, AO and SCSO algorithms have the same complexity. The proposed algorithm, only in the second term of the complexity, has a difference in coefficients with YDSE, SO, HBA, SSA, WSO, and COOT, mainly due to the introduced crossover strategy. In fact, real-world optimization problems are complex and nonlinear. Therefore, when confronted with real-world optimization problems, the complexity of the proposed FYDSE is comparable to that of YDSE, SO, HBA, SSA, WSO, COOT, and AHA and outperforms DO, AO, and SCSO and significantly outperforms ChOA. To demonstrate this conclusion more effectively, we provide the runtime results for the ten functions in the cec2022 suite in Table 5.

From the results in the table, the overall operational results of the proposed FYDSE are better than those of the ChOA, SCSO, and DO algorithms. Also, the gap between the running results of the proposed algorithm and the other algorithms is considered small. At the same time, this gap will gradually decrease as the complexity of the objective function of the problem under study increases. Therefore, the complexity of the proposed FYDSE is improved within the acceptable range, and it is important to note that it cannot be ignored that the proposed algorithm obtains better performance results in these benchmark suites.

### 4.5. Search Capability Analysis of Global Optimal Solutions

In order to verify the improvement in the introduced initialization and exploration strategies on the global solution searchability of the YDSE algorithm, we provide different methods to obtain the probability of the global optimal solution. First, considering that some test functions are difficult to converge to the optimal position, we consider that the candidate solution of the solution is considered optimal if it converges to the position of the neighborhood of the optimal solution, i.e., the relative error ((Candidate Solution − Optimal Solution)/Candidate Solution) is < 10^−2^. We provide the results of the probability of reaching the optimal solution for the twenty runs of the proposed FYDSE and YDSE, as well as the other methods, in Table 6.

The results in Table 6 show that the proposed FYDSE achieves the best probability of reaching the optimal solution for most of the test functions and has the best final average ranking. Compared with YDSE, the proposed FYDSE has a higher probability of searching for the optimal solution in cec02, cec07, cec08, and cec10. The main reason is that the probability of obtaining the global optimal solution is improved by introducing a dynamic adversarial strategy that effectively extends the full exploration of seldom-searched regions.

## 5. An Example of Complex Engineering Optimization: The Four-Stage Gearbox Problem

**Figure 7:**
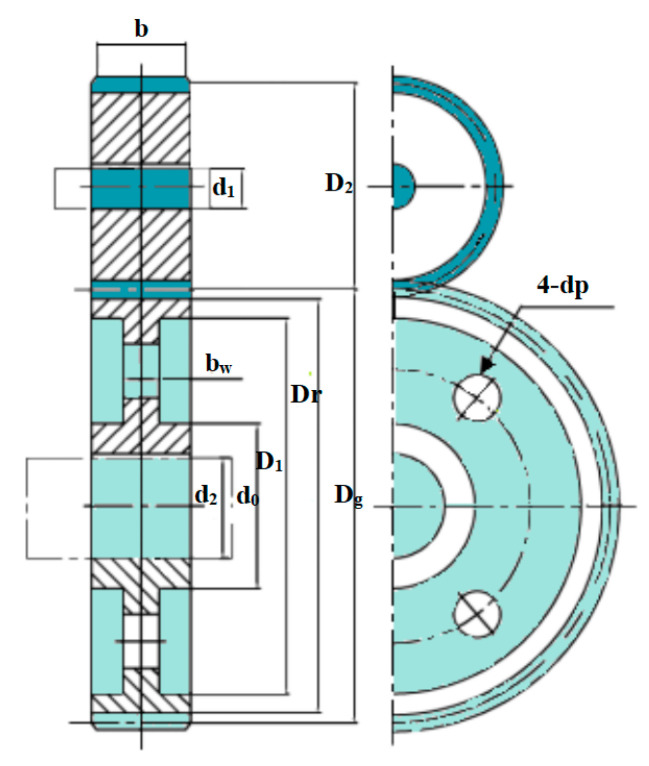
Schematic diagram of the four-stage gearbox problem.

To further validate the performance of the proposed FYDSE in solving complex engineering optimization problems, the proposed algorithm is experimentally compared with other algorithms, including the original YDSE, in a complex engineering optimization example: a four-stage gearbox problem. A schematic diagram of the four-stage gearbox problem is shown in Figure 7. The objective of the problem is to minimize the weight of the gearbox, which contains 22 discrete independent variables [58]. They are categorized into four types, including gear position, pinion position, billet thickness, and number of teeth. In addition, the problem contains 86 nonlinear design constraints related to pitch, kinematics, contact ratio, gear strength, gear assembly, and gear size [44]. The mathematical model is shown below:

Minimize:

(33) $$f(\bar{x})=\left(\frac{\pi }{1000}\right)\;\sum_{i=1}^{4}\frac{b_{i}c_{i}^{2}(N_{pi}^{2}+N_{gi}^{2})}{{\left(N_{pi}+N_{gi}\right)}^{2}},\;\;\mathrm{where},\;i=(1,2,3,4).$$

Subject to:

(34) $$g_{1}(\bar{x})=\left(\frac{366000}{\pi \omega_{1}}+\frac{2c_{1}N_{p1}}{N_{p1}+N_{g1}}\right)\left(\frac{{\left(N_{p1}+N_{g1}\right)}^{2}}{4b_{1}c_{1}^{2}N_{p1}}\right)-\frac{\sigma_{N}J_{R}}{0.0167WK_{o}K_{m}}\leq 0,$$

(35) $$g_{2}(\bar{x})=\left(\frac{366000N_{g1}}{\pi \omega_{1}N_{p1}}+\frac{2c_{2}N_{p2}}{N_{p2}+N_{g2}}\right)\left(\frac{{\left(N_{p2}+N_{g2}\right)}^{2}}{4b_{2}c_{2}^{2}N_{p2}}\right)-\frac{\sigma_{N}J_{R}}{0.0167WK_{o}K_{m}}\leq 0,$$

(36) $$g_{3}(\bar{x})=\left(\frac{366000N_{g1}N_{g2}}{\pi \omega_{1}N_{p1}N_{p2}}+\frac{2c_{3}N_{p3}}{N_{p3}+N_{g3}}\right)\left(\frac{{\left(N_{p3}+N_{g3}\right)}^{2}}{4b_{3}c_{3}^{2}N_{p3}}\right)-\frac{\sigma_{N}J_{R}}{0.0167WK_{o}K_{m}}\leq 0,$$

(37) $$g_{4}(\bar{x})=\left(\frac{366000N_{g1}N_{g2}N_{g3}}{\pi \omega_{1}N_{p1}N_{p2}N_{p3}}+\frac{2c_{4}N_{p4}}{N_{p4}+N_{g4}}\right)\left(\frac{{\left(N_{p4}+N_{g4}\right)}^{2}}{4b_{4}c_{4}^{2}N_{p4}}\right)-\frac{\sigma_{N}J_{R}}{0.0167WK_{o}K_{m}}\leq 0,$$

(38) $$g_{5}(\bar{x})=\left(\frac{366000}{\pi \omega_{1}}+\frac{2c_{1}N_{p1}}{N_{p1}+N_{g1}}\right)\left(\frac{{\left(N_{p1}+N_{g1}\right)}^{3}}{4b_{1}c_{1}^{2}N_{g1}N_{p1}^{2}}\right)-{\left(\frac{\sigma_{H}}{C_{p}}\right)}^{2}\left(\frac{\sin(\varphi )\cos(\varphi )}{0.0334WK_{o}K_{m}}\right)\leq 0,$$

(39) $$g_{6}(\bar{x})=\left(\frac{366000N_{g1}}{\pi \omega_{1}N_{p1}}+\frac{2c_{2}N_{p2}}{N_{p2}+N_{g2}}\right)\left(\frac{{\left(N_{p2}+N_{g2}\right)}^{3}}{4b_{2}c_{2}^{2}N_{g2}N_{p2}^{2}}\right)-{\left(\frac{\sigma_{H}}{C_{p}}\right)}^{2}\left(\frac{\sin(\varphi )\cos(\varphi )}{0.0334WK_{o}K_{m}}\right)\leq 0,$$

(40) $$g_{7}(\bar{x})=\left(\frac{366000N_{g1}N_{g2}}{\pi \omega_{1}N_{p1}N_{p2}}+\frac{2c_{3}N_{p3}}{N_{p3}+N_{g3}}\right)\left(\frac{{\left(N_{p3}+N_{g3}\right)}^{3}}{4b_{3}c_{3}^{2}N_{g3}N_{p3}^{2}}\right)-{\left(\frac{\sigma_{H}}{C_{p}}\right)}^{2}\left(\frac{\sin(\varphi )\cos(\varphi )}{0.0334WK_{o}K_{m}}\right)\leq 0,$$

(41) $$g_{8}(\bar{x})=\left(\frac{366000N_{g1}N_{g2}N_{g3}}{\pi \omega_{1}N_{p1}N_{p2}N_{p3}}+\frac{2c_{4}N_{p4}}{N_{p4}+N_{g4}}\right)\left(\frac{{\left(N_{p4}+N_{g4}\right)}^{3}}{4b_{4}c_{4}^{2}N_{g4}N_{p4}^{2}}\right)-{\left(\frac{\sigma_{H}}{C_{p}}\right)}^{2}\left(\frac{\sin(\varphi )\cos(\varphi )}{0.0334WK_{o}K_{m}}\right)\leq 0,$$

(42) $$\begin{array}{l} g_{9-12}(\bar{x})=-N_{pi}\sqrt{\frac{\sin^{2}(\varphi )}{4}-\frac{1}{N_{pi}}+{\left(\frac{1}{N_{pi}}\right)}^{2}}+N_{gi}\sqrt{\frac{\sin^{2}(\varphi )}{4}+\frac{1}{N_{pi}}{\left(\frac{1}{N_{pi}}\right)}^{2}}+\\ \;\;\;\;\frac{\sin(\varphi )(N_{pi}+N_{gi})}{2}+CR_{\min}\pi \cos(\varphi )\leq 0, \end{array}$$

(43) $$g_{13-16}(\bar{x})=d_{\min}-\frac{2c_{i}N_{pi}}{N_{pi}+N_{gi}}\leq 0,$$

(44) $$g_{17-20}(\bar{x})=d_{\min}-\frac{2c_{i}N_{gi}}{N_{pi}+N_{gi}}\leq 0,$$

(45) $$g_{21}(\bar{x})=x_{p1}+\left(\frac{\left(N_{p1}+2\right)c_{1}}{N_{p1}+N_{g1}}\right)-L_{\max}\leq 0,$$

(46) $$g_{22-24}(\bar{x})=-L_{\max}+{\left(\frac{\left(N_{pi}+2\right)c_{i}}{N_{pi}+N_{gi}}\right)}_{i=2,3,4}+x_{g(i-1)}\leq 0,$$

(47) $$g_{25}(\bar{x})=-x_{p1}+\frac{\left(N_{p1}+2\right)c_{1}}{N_{p1}+N_{g1}}\leq 0,$$

(48) $$g_{26-28}(\bar{x})={\left(\frac{\left(N_{pi}+2\right)c_{i}}{N_{pi}+N_{gi}}-x_{g(i-1)}\right)}_{i=2,3,4}\leq 0,$$

(49) $$g_{29}(\bar{x})=y_{p1}+\frac{\left(N_{p1}+2\right)c_{1}}{N_{p1}+N_{g1}}-L_{\max}\leq 0,$$

(50) $$g_{30-32}(\bar{x})=-L_{\max}+{\left(\frac{c_{i}\left(2+N_{pi}\right)}{N_{pi}+N_{gi}}+y_{g(i-1)}\right)}_{i=2,3,4}\leq 0,$$

(51) $$g_{33}(\bar{x})=\frac{\left(2+N_{p1}\right)c_{1}}{N_{p1}+N_{g1}}-y_{p1}\leq 0,$$

(52) $$g_{34-36}(\bar{x})={\left(\frac{c_{i}\left(2+N_{pi}\right)}{N_{pi}+N_{gi}}-y_{g(i-1)}\right)}_{i=2,3,4}\leq 0,$$

(53) $$g_{37-40}(\bar{x})=-L_{\max}+\frac{c_{i}\left(2+N_{gi}\right)}{N_{pi}+N_{gi}}+x_{gi}\leq 0,$$

(54) $$g_{41-44}(\bar{x})=-x_{gi}+\left(\frac{\left(N_{g1}+2\right)c_{i}}{N_{pi}+N_{gi}}\right)\leq 0,$$

(55) $$g_{45-48}(\bar{x})=y_{gi}+\left(\frac{\left(N_{gi}+2\right)c_{i}}{N_{pi}+N_{gi}}\right)-L_{\max}\leq 0,$$

(56) $$g_{49-52}(\bar{x})=-y_{gi}+\left(\frac{\left(N_{gi}+2\right)c_{i}}{N_{pi}+N_{gi}}\right)\leq 0,$$

(57) $$g_{53-56}(\bar{x})=(b_{i}-8.255)(b_{i}-5.715)(b_{i}-12.70)(-N_{pi}+0.945c_{i}-N_{gi})(-1)\leq 0,$$

(58) $$g_{57-60}(\bar{x})=(b_{i}-8.255)(b_{i}-3.175)(b_{i}-12.70)(-N_{pi}+0.646c_{i}-N_{gi})\leq 0,$$

(59) $$g_{61-64}(\bar{x})=(b_{i}-5.715)(b_{i}-3.175)(b_{i}-12.70)(-N_{pi}+0.504c_{i}-N_{gi})\leq 0,$$

(60) $$g_{65-68}(\bar{x})=(b_{i}-5.715)(b_{i}-3.175)(b_{i}-8.255)(0c_{i}-N_{pi}-N_{gi})\leq 0,$$

(61) $$g_{69-72}(\bar{x})=(b_{i}-8.255)(b_{i}-5.715)(b_{i}-12.70)(N_{pi}+N_{gi}-1.812c_{i})(-1)\leq 0,$$

(62) $$g_{73-76}(\bar{x})=(b_{i}-8.255)(b_{i}-3.175)(b_{i}-12.70)(-0.945c_{i}+N_{pi}+N_{gi})\leq 0,$$

(63) $$g_{77-80}(\bar{x})=(b_{i}-5.715)(b_{i}-3.175)(b_{i}-12.70)(-0.646c_{i}+N_{pi}+N_{gi})(-1)\leq 0,$$

(64) $$g_{81-84}(\bar{x})=(b_{i}-5.715)(b_{i}-3.175)(b_{i}-8.255)(N_{pi}+N_{gi}-0.504c_{i})\leq 0,$$

(65) $$g_{85}(\bar{x})=\omega_{\min}-\frac{\omega_{1}(N_{p1}N_{p2}N_{p3}N_{p4})}{N_{g1}N_{g2}N_{g3}N_{g4}}\leq 0,$$

(66) $$g_{86}(\bar{x})=\frac{\omega_{1}(N_{p1}N_{p2}N_{p3}N_{p4})}{N_{g1}N_{g2}N_{g3}N_{g4}}-\omega_{\max}\leq 0,$$

where:

(67) $$\bar{x}=\left\{N_{p1},N_{g1},N_{p2},N_{g2}\cdots b_{1},b_{2}\cdots x_{p1},x_{g1},x_{p2},x_{g2}\cdots y_{p1},y_{g1},y_{p2},y_{g2}\cdots y_{g4}\right\}\;,\;c_{i}=\sqrt{{\left(y_{gi}-y_{pi}\right)}^{2}+{\left(x_{gi}-x_{pi}\right)}^{2}},K_{o}=1.5,d_{\min}=25,J_{R}=0.2,\;\varphi =120,W=55.9,K_{M}=1.6,CR_{\min}=1.4,L_{\max}=127,C_{p}=464,\;\sigma_{H}=3290,\omega_{\max}=255,\omega_{1}=5000,\sigma_{N}=2090,\omega_{\min}=245,$$

With bounds:

(68) $$b_{i}\in \{3.175,12.7,8.255,5.715\},\;y_{pi},x_{pi},y_{gi},x_{gi}\in \{12.7,38.1,25.4,50.8,76.2,63.5,88.9,114.3,101.6\},\;7\leq N_{gi},N_{pi}\leq 76\in$$

**Figure 8:**
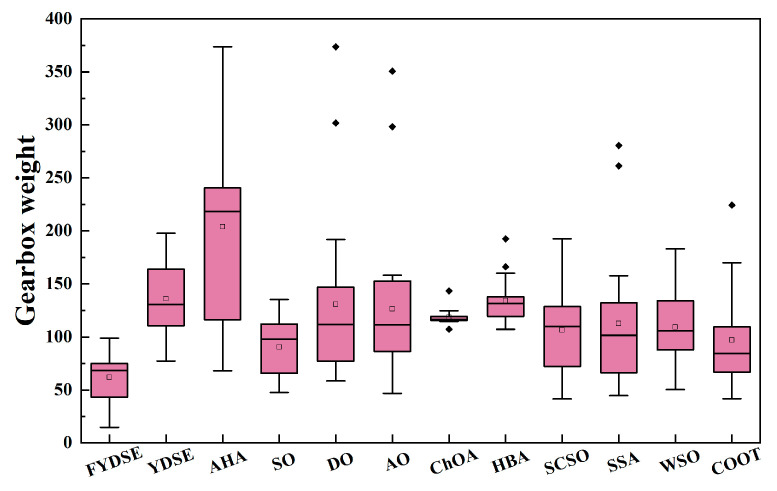
Box plots of gearbox weights for FYDSE and other methods at 20 runs.

Table 7 gives the minimum, worst, and average gearbox weights and values of the design variables obtained after 20 runs of the proposed FYDSE with YDSE, AHA, SO, DO, AO, ChOA, HBA, SCSO, SSA, WSO, and COOT. Also, the running times of all the methods are given at the end of the table. The gearbox weight box plots for FYDSE and other methods at 20 runs are given in Figure 8. From the results, the difference between the running time of the proposed FYDSE method and YDSE is small when facing complex optimization problems due to the high complexity of the objective function. Also, the proposed algorithm obtains the best minimum and average gearbox weight with a significant difference compared to other methods and YDSE. Therefore, the introduced four strategies can effectively improve the algorithm’s ability to face complex engineering and realistic optimization problems.

## 6. Specific Truss Topology Optimization Problems (TTOP)

To validate the efficacy of FYDSE in tackling intricate engineering optimization challenges, it is employed to optimize the topology of the truss structure. Compared with the cross-section optimization of trusses, topology optimization attempts to streamline the mass of the truss by eliminating unnecessary members and nodes. Therefore, topology optimization can effectively reduce the cost when the cost of nodes is large. In our implementation, we impose frequency constraints on the intrinsic frequencies to ensure that they are effectively limited. Conventional optimization techniques, including sensitivity analysis, have garnered widespread application in addressing truss optimization challenges and have yielded notable outcomes. However, the efficacy of traditional methods in tackling intricate optimization challenges remains in need of enhancement. Meta-heuristic algorithms are an effective solution to this problem since they can fully explore the nonlinear and nonconvex space of topological optimization and maintain the topological results. Therefore, in this section, the efficacy of the FYDSE algorithm in addressing highly nonlinear and nonconvex TTOP problems is further validated. Moving forward, we present the mathematical model for topology optimization.

### 6.1. TTOP Model

The primary goal of the TTOP was to ascertain the best truss structure and layout to ensure that the ground structure could carry the minimum possible loads. This ground structure comprises both necessary and discretionary nodes. Necessary nodes are typically regarded as promptly bearing structural, load, and nodal stresses [59]. Conversely, including discretionary nodes enhances the stress distribution among the various components. To embark on this endeavor, we first define the key constraints and objective functions of the TTOP model.

Suppose variable *X*= {*A*_1_, *A*_2_, …, *A_p_*} and the target function of TTOP is formulated as follows:

(69) $$F(A)=\sum_{i=1}^{p}B_{i}A_{i}\rho_{i}{Length}_{i}+\sum_{j=1}^{q}b_{j},B_{i}=\{\begin{array}{c} 0,if\;A_{i}<\mathrm{Critical}\;\mathrm{region}\\ 1,if\;A_{i}\geq \mathrm{Critical}\;\mathrm{region} \end{array}$$

where *p* means numbers of variables and *q* means numbers of nodes. *ρ_i_*, *Length_i_*, *A_i_* signifies the elemental density, length, and transverse area of the *i*th variable. *b_j_* denotes the quantity of the *j*th node. We set a critical region with a small positive value to determine which elements to discard [60]. When the cross-sectional area of an element falls below a certain minimal threshold, it becomes necessary to discard it.

Further, we give multiple constraints for the TTOP problem, including stress, displacement, Euler buckling, intrinsic frequency, and upper and lower cross-section constraints. The specific definitions are shown below:

(70) $$\sigma_{i}^{\min}\leq B_{i}\sigma_{i}\leq \sigma_{i}^{\max}$$

(71) $$\delta_{j}^{\min}\leq \delta_{j}\leq \delta_{j}^{\max}$$

(72) $$-C_{i}\sigma_{i}\leq -\sigma_{i}^{E},\sigma_{i}^{E}=\frac{-k_{i}A_{i}E}{L_{i}^{2}}$$

(73) $$f_{r}^{\min}-f_{r}\leq 0$$

(74) $$A_{k}^{\min}\leq A_{k}\leq A_{k}^{\max}$$

where *σ_i_* implies the stress of the ith node. $\sigma_{i}^{\max}$ and $\sigma_{i}^{\min}$ represent the upper and lower boundary for stress constraints. *δ_j_* means the displacement of the *i*th knot. $\delta_{j}^{\max}$ and $\delta_{j}^{\min}$ represent the upper and lower boundary for displacement constraints, respectively. *fr* means the intrinsic frequency of the truss structure in *r*th mode. In addition, $A_{k}^{\min}$ and $A_{k}^{\max}$ denote the scope of variables, respectively.

In order for the variables to satisfy the above constraints, we introduce a penalty term in the objective function [61]. As illustrated in Equation (39), when the constraints are obeyed, the penalty term will be reduced to zero. However, a positive penalty will be imposed in the event of a constraint violation. The following outlines the specific penalty function in detail:

(75) $$F(A)=\{\begin{array}{c} 10^{9},\;\;\;\;\;\;\;\;\;\;\;if\;\;\;\mathrm{structure}\;\mathrm{is}\;\mathrm{not}\;\mathrm{satisfied}\;\;\;\;\;\;\;\;\;\;\;\;\;\\ 10^{8},\;\;\;\;\;\;\;\;\;\;\;if\;\;\;\mathrm{degree}\;\mathrm{of}\;\mathrm{freedom}\;\mathrm{is}\;\mathrm{not}\;\mathrm{satisfied}\;\;\;\;\;\;\;\;\\ 10^{7},\;\;\;\;\;\;\;\;\;\;\;\;\;\;\;if\;\;\;\mathrm{positive}\;\mathrm{definiteness}\;\mathrm{is}\;\mathrm{not}\;\mathrm{satisfied}\;\;\;\;\;\;\\ F(A)\cdot penalty,\;\;\;\;\;\;\;\;\;\mathrm{otherwise}\;\;\;\;\;\;\;\;\;\;\;\;\;\;\;\;\;\;\;\;\;\;\;\;\;\;\;\;\;\;\;\;\;\;\;\;\;\;\;\;\;\;\;\;\;\;\;\;\;\;\;\;\;\;\;\; \end{array}$$

where the *penalty* term is set to zero if the independent variable adheres to the specified constraint. *F*(*A*) means the weighted objective function. However, if the constraint is violated, a positive penalty is imposed. The precise formulation of this penalty function is detailed as follows:

(76) $$Penalty={\left(1+\varepsilon \cdot \sum_{i=1}^{ac}\left|1-\frac{g_{i}}{g_{i}^{*}}\right|\right)}^{\varepsilon }.$$

where *g* denotes the tendency of a variable to violate a constraint and be subject to a $g_{i}^{*}$, *ac* representation of active restraint. *ε* is set to 2 [62].

In order to fully validate the performance and usefulness of FYDSE in solving the optimized TTOPs for complex projects, three TTOPs with different numbers of trusses are discussed in this section. In these three TTOPs, all independent variables are considered to be continuous, and the Euler buckling coefficients and nodal masses are fixed to 4 kg and 5 kg. The cross-section size range is set between *A*_max_ and −*A*_max_. To fairly assess the engineering optimization capabilities of FYDSE, we compare it with the original YDSE as well as other latest optimization algorithms, including AHA, SO, DO, AO, ChOA, HBA, SCSO, SSA, WSO, and COOT. To ensure the fairness and repeatability of the optimization TTOP, we made the population size of all algorithms 30 with a maximum iteration of 1000. In addition, all algorithms are tested with 30 independent repetitions.

### 6.2. 20-Truss Topology Optimization

**Figure 9:**
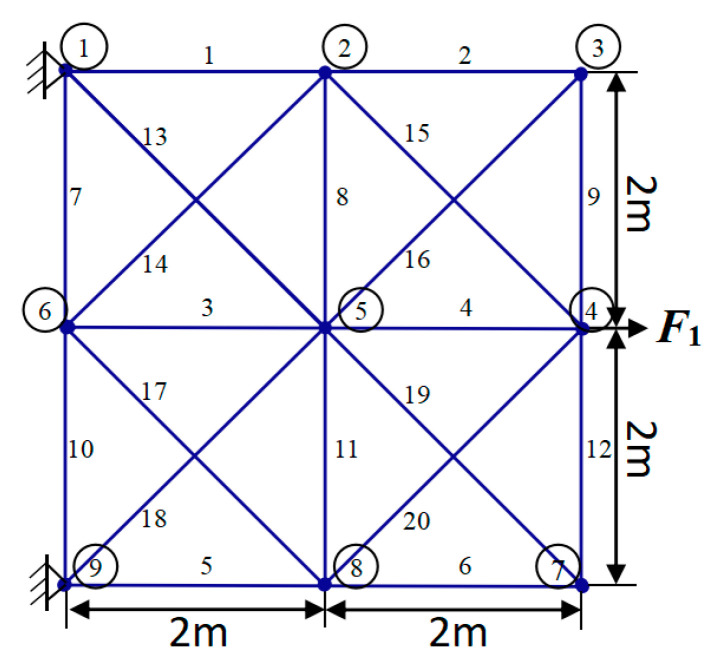
Schematic diagram of 20-rod truss structures.

Figure 9 illustrates the initial schematic ground architecture of a 20-rod truss consisting of 20 members and 9 knots. In this figure, all 20 members and 9 nodes are clearly labeled. Of particular note is that the weights of this truss structure are mainly carried by the supports of node 1 and node 9.

Furthermore, to enhance the optimization of this truss structure, we have compiled a comprehensive list of detailed constraints and inherent material properties in Table 8. Additionally, Table 9 comprehensively displays the experimental outcomes obtained by various comparative methods in addressing the 20-truss optimization problem. Among the experimental results, according to a model introduced for TTOP, we further optimized the design of the truss structure by deleting some unwanted parts (indicated by “-”) according to the requirements.

The outcomes of the independent variables and objective functions of YDSE, AHA, SO, DO, AO, ChOA, HBA, SCSO, SSA, WSO, COOT and the proposed FYDSE are given in Table 9. Upon examination of the research results outlined in Table 9, it is evident that the FYDSE and the AHA, HBA, and COOT all attained the desired minimum optimal weight, measuring precisely 154.799. However, among these algorithms, the proposed FYDSE algorithm shows its unique advantage, with an average weight of only 164.718, significantly smaller than the average weights of other algorithms. In contrast, the SSA and SO algorithms ranked second and third, with their optimal structural qualities of 155.347 and 155.574, which failed to surpass the performance of the FYDSE algorithm despite their proximity. This result indicates that the FYDSE algorithm has higher efficiency and stability in weight optimization.

**Figure 10:**
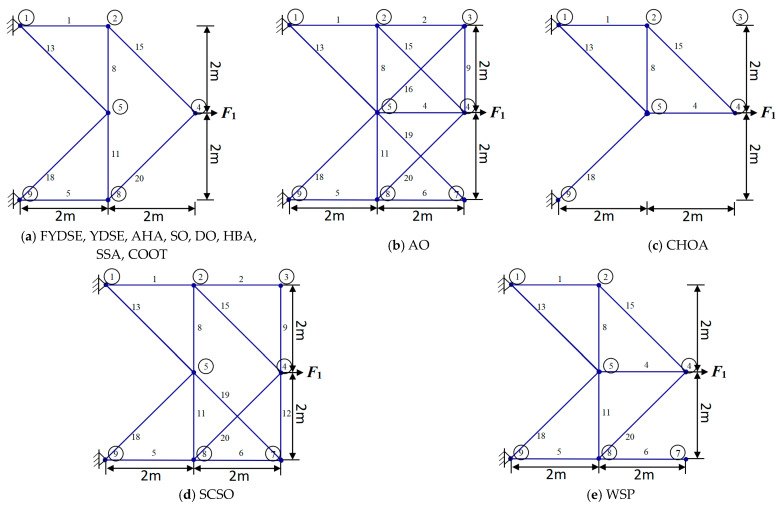
FYDSE and comparison algorithms for 20-bar truss topology optimization structures.

Figure 10 illustrates the optimal topology achieved through the FYDSE method and a comparative analysis with alternative algorithms during optimization. Specifically, all eight algorithms, FYDSE, YDSE, AHA, SO, DO, HBA, SSA, and COOT, select eight members as the basis of the topology, and these members have the same numbering in their respective algorithms. However, the CHOA algorithm employs a different strategy for retaining only six members. Although the CHOA algorithm requires a more streamlined component structure than that suggested by the FYDSE algorithm, it is more stringent in terms of the quality of the structure. As shown in Figure 10a, YDSE, AHA, SO, DO, HBA, SSA, COOT, and FYDSE present consistency in topology, and their main difference lies in the fact that there are different sizes of cross-sections in the structure. These algorithms provide a valuable reference for solving practical problems, especially when applying metaheuristics. It is worth mentioning that our proposed FYDSE algorithm shows excellent performance in solving a topology optimization problem containing 20 trusses. In contrast to other SOTA algorithms, FYDSE performs better in terms of optimization efficiency and quality of results, further validating its effectiveness and usefulness in topology optimization.

### 6.3. 24-Truss Topology Optimization

**Figure 11:**
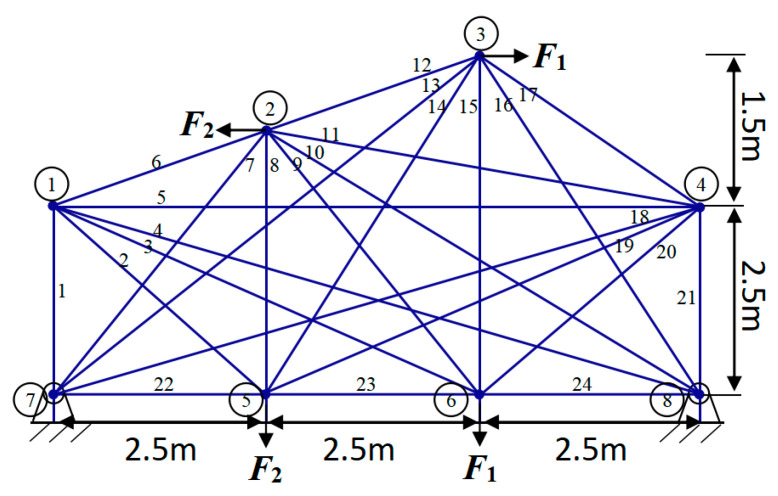
Schematic diagram of 24-rod truss structures.

Figure 11 shows a structural diagram for an original ground configuration of a 24-rod truss consisting of 24 members and 8 knots, all clearly labeled. Notably, node 3 supports a non-structural concentrated mass of 500 kg. While this additional weight does not contribute to the overall weight of the truss, it significantly influences the design considerations for the positioning and dimensions of both nodes and members.

To facilitate the optimization process for the 24-rod truss structure, we have compiled a list of pertinent constraints and the inherent material properties in Table 10. Moreover, the results of comparing the method with FYDSE in terms of independent variables and minimum weight is presented in Table 11. Among the experimental results, according to the introduction of the TTOP model, we further optimized the design of the truss structure by deleting some unwanted parts (indicated by “-”) according to the requirements.

The consequences of the independent variables and objective functions of YDSE, AHA, SO, DO, AO, ChOA, HBA, SCSO, SSA, WSO, COOT and the proposed FYDSE are shown in Table 11. Based on the results of the experiment, we can see that the HBA, COOT, and our proposed FYDSE algorithms achieve the same and the lowest result, i.e., 126.252, in seeking the optimal weights, and none of them violates the set constraints. However, among these algorithms, the proposed FYDSE algorithm shows its unique advantage, with an average weight of only 139.951, significantly smaller than the average weights of other algorithms. In contrast, the HBA and COOT algorithms positioned themselves in the second and third spots, respectively, boasting optimal structural qualities of 178.958 and 146.784, which failed to surpass the performance of the FYDSE algorithm despite their proximity. The FYDSE algorithm performs optimally in terms of average weights, a result that further proves the stability and reliability of the FYDSE algorithm.

**Figure 12:**
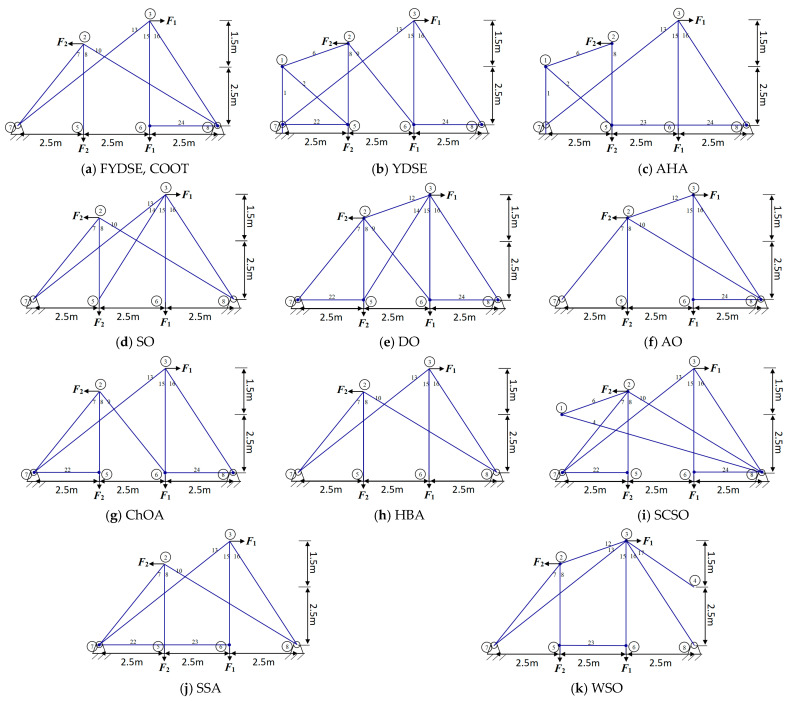
FYDSE and comparison algorithms for 24-rod truss topology optimization structures.

In Figure 12, a comparative analysis is presented, showcasing the optimal topology attained by the FYDSE algorithm in contrast to that of other algorithms. FYDSE, COOT, SO, AO, and SSA successfully retain seven building blocks among these algorithms. FYDSE and COOT corresponded to the same number of building blocks, while SO, AO, and SSA chose different numbers. Although the HBA algorithm retains only six building blocks, this may imply that it is more stringent regarding structural quality. The topology of the FYDSE algorithm is more reasonable regarding layout and connections looking at the structure of the figure. When dealing with the problem of topology optimization with a 24-rod truss, our proposed FYDSE algorithm outperforms other recent methods.

### 6.4. 72-Truss Topology Optimization

**Figure 13:**
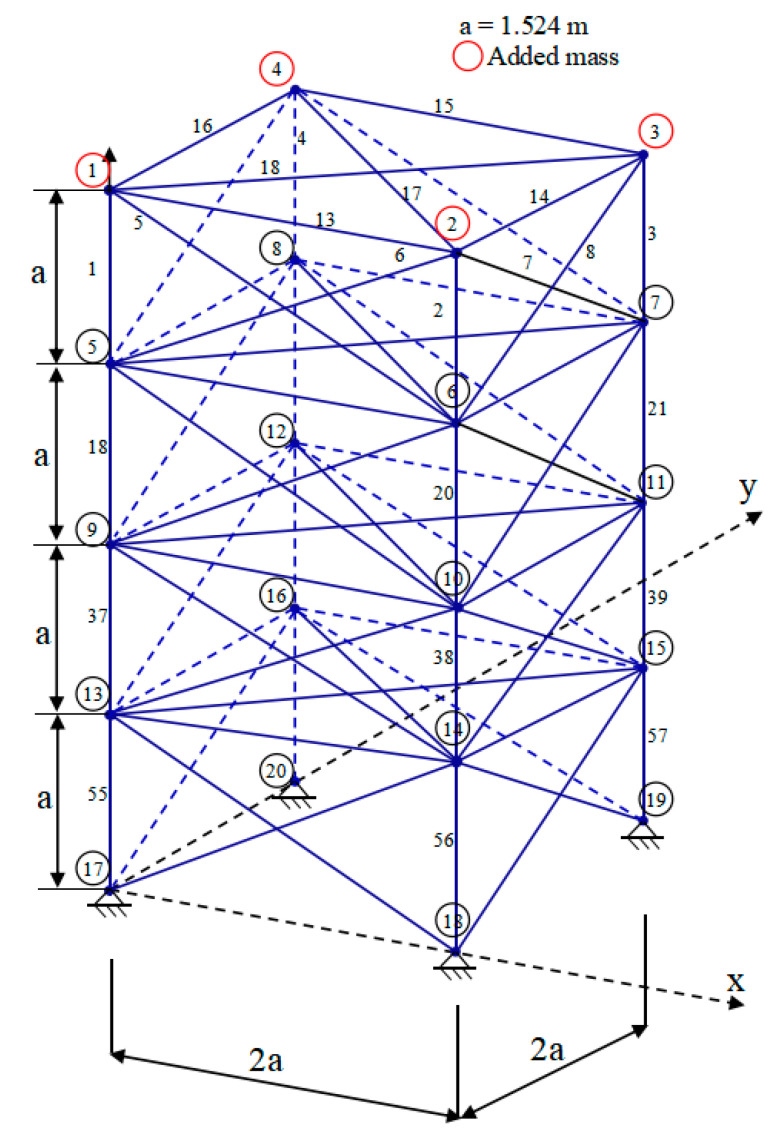
Schematic diagram of 72-rod truss structure.

Figure 13 comprehensively illustrates the original ground structure schematic of a 72-rod truss comprising 72 members and 20 knots, with select members and nodes labeled for clarity. It is crucial to consider the impact of the center on the positioning and dimensions of these members and knots when determining the ultimate total weight of this 72-rod truss structure.

In order to optimize this truss structure, we have detailed the materials’ associated constraints and inherent properties in Table 12.

In addition, the results of comparing the method with FYDSE in terms of independent variables and minimum weight are presented in Table 13. Among the experimental results, according to the introduction of the TTOP model, we further optimized the design of the truss structure by deleting some unwanted parts (indicated by “-”) according to the requirements.

Drawing from the experimental data presented in Table 13, we observe that the SCSO, COOT, and our proposed FYDSE algorithm attain the lowest optimal weight value of 450.388. Nevertheless, the FYDSE algorithm stands out in its performance, exhibiting a significantly lower average weight value of 454.209, surpassing all comparable algorithms. These data further highlight the stability and reliability of the FYDSE algorithm. Among the comparison algorithms, the SO algorithm also performs exceptionally well, with an average weight of 458.213, the second highest, and its structural quality is equally satisfactory.

**Figure 14:**
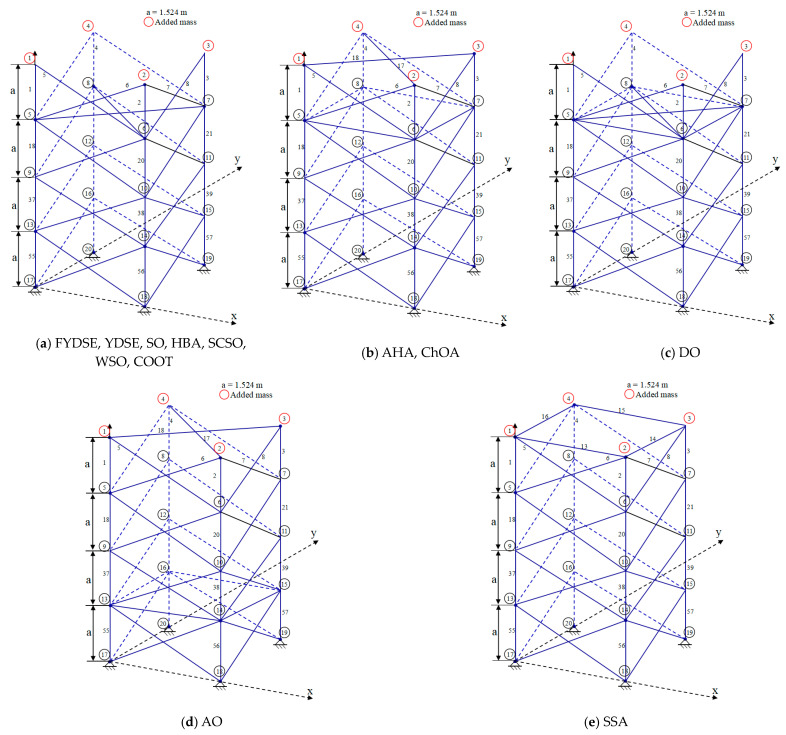
FYDSE and comparison algorithms for 72-rod truss topology optimization structures.

To have a more intuitive understanding of the optimization effect of these algorithms, Figure 14 exhibits the optimal topologies derived through FYDSE and its comparative algorithms [63,64]. Figure 14a indicates that the YDSE, SO, HBA, SCSO, WSO, COOT, and FYDSE algorithms retain the same number of building blocks during optimization. In addition, the AHA and ChOA algorithms also retain the same number of building blocks. These algorithms remain consistent in the number of building blocks and labeling, but the cross-sectional area in the topology varies in size. In addressing the optimization challenge of truss topology, our FYDSE surpasses the performance of other cutting-edge algorithms, exhibiting remarkable efficiency and effectiveness. It proves once again its superiority in the field of optimal structural design.

## 7. Discussion

By comparing the results in this study with other methods, the proposed FYDSE shows excellent optimization performance. Based on two experiments, the advantages of FYDSE are specifically discussed. The first part compares FYDSE with other methods on the CEC2022 test function. Better results are obtained on 11 functions compared to YDSE, demonstrating that the introduction of the four strategies sufficiently improves the algorithmic shortcomings of YDSE. Meanwhile, the FYDSE algorithm optimized eight functions, which accounted for 66.6% of all functions. This result shows that the fractional-order modified mechanism, dynamic opposition, and vertical crossover operator enhance the effective search and comprehensive utilization of YDSE, which confirms that FYDSE optimizes the test problem with higher accuracy, better reliability, and faster conversion speed. The second part discusses the performance of FYDSE with other methods in three truss topology optimizations. FYDSE has the best average and optimal weights in all three cases. The results validate the effectiveness and reliability of FYDSE in engineering optimization. Therefore, the advantages of FYDSE in solving numerical and engineering optimization problems are summarized as follows:

(1) FYDSE ranks better than the original YDSE in most CEC2022 tests. The improved strategy introduced by FYDSE can efficiently balance convergence speed and convergence accuracy and search for better solutions.
(2) In the CEC2022 test, FYDSE outperforms other algorithms, which shows that FYDSE has a smooth development and exploration process, and is not easily trapped in localized solutions when facing multi-type problems.
(3) The optimal average weights and minimum weights of FYDSE in the three truss topology optimization indicate the effectiveness, reliability, and stability of FYDSE in solving engineering optimization problems.

## 8. Conclusions

In order to solve the problems that the original YDSE will easily fall into local optimums, converge slowly, and have an imbalance between exploration and exploitation when dealing with sophisticated engineering optimal problems, this paper presents a fractional-order boosted hybrid YDSE for solving complex optimization problems. FYDSE introduces a piecewise chaotic mapping strategy, a fractional-order improvement strategy, a dynamic dyadic strategy, and a vertical operator. First, the fractional-order strategy is introduced in the dark edge position update of FYDSE to ensure that the search potential of a single neighborhood space is exploited more efficiently while decreasing the likelihood of trapping in a local optimum. Secondly, during the initialization phase, the incorporation of piecewise chaotic mapping is intended to yield a high-quality primary population, thereby enhancing algorithm convergence efficiency. Furthermore, the low exploration space of FYDSE is extended by using a dynamic inversion strategy, which improves the probability of obtaining a globally optimal solution. Finally, by introducing the vertical operator, FYDSE can better balance global exploration and local exploitation and explore new unknown areas. Comparative experiments of FYDSE with a series of state-of-the-art algorithms are conducted in the CEC2022 test suite, the four-stage gearbox problem, and three TTOP cases, respectively. The Wilcoxon rank-sum test statistically proves the effectiveness of FYDSE. In addition, the average ranking of the mean result and probability of FYDSE in cec2022 are 1.42 and 1.33, respectively, verifying that FYDSE can better converge to the optimal solution. Although FYDSE increases the constant complexity by introducing improvement strategies, this difference is small when facing complex problems as analyzed through experiments. The efficient performance of the four-stage gearbox problem and the three TTOP cases verifies that FYDSE is an effective method for solving complex engineering optimization challenges.

Many complex, nonlinear, and high-dimensional realistic optimization problems, such as complex path planning for UAVs, neural network parameter optimization, and curved shape parameter optimization problems, are yet to be solved in scientific and practical research. The better experimental results for numerical and engineering optimization problems show that the proposed FYDSE is an optimization method that can be extended in different fields. Therefore, FYDSE can be used as a potential solution to solve various complex real-world problems effectively.

As a direction for future work, we will strive to extend FYDSE to other domain-specific optimization problems, such as UAV path planning and image threshold segmentation. Moreover, we will also explore the integration of FYDSE with machine learning and deep learning techniques, such as using FYDSE to solve feature selection problems or for deep learning parameter optimization.

## Figures and Tables

**Table 1:** Configuring algorithm parameters, where the parameter settings for the comparison experiments are all referenced to the results provided in the original references.

Algorithms	Year	Parameter Setting
YDSE	2023	Wavelength (*λ*) = 5 × 10^−6^ m, distance between two slits (*d*) = 5 × 10^−3^ m, distance between the barrier and the projection screen (*L*) = 1 m, distance between light source and barrier (*I*) = 0.01 m, constant value (*δ*) = 0.38.
AHA	2022	Migration coefficient = 2*n*.
DO	2022	α = [0, 1], *k* = [0, 1].
ChOA	2020	*m* is chaotic.
HBA	2022	*β* (the ability of a honey badger to get food) = 6, *C* = 2.
SCSO	2022	Sensitivity range (*r_G_*) = [2, 0], phases control range (*R*) = [−2*r_G_*, 2*r_G_*].
SSA	2020	*ST* = 0.8.
WSO	2022	*f*_min_ = 0.07, *f*_max_ = 0.75, *τ* = 4.125, *a*_0_ = 6.25, *a*_1_ = 100, *a*_2_ = 0.0005.

**Table 2:** Results of FYDSE and various comparative methods on cec2022; the optimal value is in bold.

Function	Index	Algorithms											
		YDSE	AHA	SO	DO	AO	ChOA	HBA	SCSO	SSA	WSO	COOT	FYDSE
cec01	Best	3.00E+02	3.00E+02	3.00E+02	3.00E+02	6.58E+02	8.39E+02	3.00E+02	3.10E+02	3.00E+02	3.00E+02	3.00E+02	3.00E+02
	Worst	3.00E+02	3.03E+02	3.06E+02	3.00E+02	6.03E+03	4.52E+03	3.00E+02	5.24E+03	3.00E+02	1.10E+03	3.00E+02	3.00E+02
	Mean	3.00E+02	3.00E+02	3.01E+02	3.00E+02	1.92E+03	2.25E+03	**3.00E+02**	1.64E+03	**3.00E+02**	4.60E+02	3.00E+02	3.00E+02
	Std	2.37E−04	5.96E−01	1.48E+00	8.21E−03	1.50E+03	1.21E+03	8.16E−06	1.65E+03	7.41E−10	2.11E+02	2.33E−02	5.38E−05
	Rank	4	7	8	5	11	12	2	10	1	9	6	3
cec02	Min	4.00E+02	4.00E+02	4.00E+02	4.00E+02	4.02E+02	4.34E+02	4.00E+02	4.00E+02	4.04E+02	4.00E+02	4.00E+02	4.00E+02
	Max	4.09E+02	4.71E+02	4.64E+02	4.75E+02	4.53E+02	2.05E+03	4.09E+02	4.74E+02	4.09E+02	4.88E+02	4.90E+02	4.00E+02
	Mean	4.04E+02	4.06E+02	4.06E+02	4.21E+02	4.19E+02	7.28E+02	4.04E+02	4.26E+02	4.06E+02	4.12E+02	4.17E+02	**4.00E+02**
	Std	2.50E+00	1.57E+01	1.40E+01	3.06E+01	1.47E+01	3.64E+02	2.86E+00	2.52E+01	1.59E+00	2.71E+01	2.90E+01	2.00E−02
	Rank	2	4	5	10	9	12	3	11	6	7	8	1
cec03	Min	6.01E+02	6.00E+02	6.00E+02	6.00E+02	6.05E+02	6.13E+02	6.00E+02	6.04E+02	6.01E+02	6.00E+02	6.00E+02	6.00E+02
	Max	6.06E+02	6.09E+02	6.06E+02	6.20E+02	6.39E+02	6.48E+02	6.01E+02	6.37E+02	6.37E+02	6.10E+02	6.08E+02	6.01E+02
	Mean	6.02E+02	6.01E+02	6.01E+02	6.05E+02	6.17E+02	6.29E+02	**6.00E+02**	6.17E+02	6.09E+02	6.02E+02	6.03E+02	6.00E+02
	Std	1.20E+00	2.01E+00	1.37E+00	4.50E+00	8.86E+00	8.69E+00	3.45E−01	8.36E+00	9.13E+00	2.80E+00	2.61E+00	1.57E−01
	Rank	6	3	4	8	11	12	1	10	9	5	7	2
cec04	Min	8.05E+02	8.16E+02	8.07E+02	8.15E+02	8.12E+02	8.20E+02	8.07E+02	8.10E+02	8.07E+02	8.03E+02	8.08E+02	8.04E+02
	Max	8.24E+02	8.39E+02	8.22E+02	8.55E+02	8.33E+02	8.48E+02	8.24E+02	8.34E+02	8.55E+02	8.18E+02	8.27E+02	8.18E+02
	Mean	8.13E+02	8.25E+02	8.16E+02	8.33E+02	8.22E+02	8.38E+02	8.18E+02	8.25E+02	8.28E+02	**8.07E+02**	8.15E+02	8.11E+02
	Std	4.98E+00	6.74E+00	4.51E+00	1.13E+01	5.52E+00	7.75E+00	4.10E+00	7.71E+00	1.22E+01	3.56E+00	4.96E+00	3.60E+00
	Rank	3	8	5	11	7	12	6	9	10	1	4	2
cec05	Min	9.00E+02	9.00E+02	9.00E+02	9.00E+02	9.35E+02	1.09E+03	9.00E+02	9.23E+02	9.00E+02	9.00E+02	9.00E+02	9.00E+02
	Max	9.17E+02	1.14E+03	1.04E+03	1.51E+03	1.33E+03	1.62E+03	9.53E+02	1.38E+03	1.29E+03	1.07E+03	1.01E+03	9.18E+02
	Mean	**9.03E+02**	9.37E+02	9.22E+02	1.01E+03	1.02E+03	1.28E+03	9.08E+02	1.12E+03	9.20E+02	9.28E+02	9.11E+02	9.04E+02
	Std	3.65E+00	5.55E+01	3.24E+01	1.90E+02	9.30E+01	1.59E+02	1.66E+01	1.43E+02	8.69E+01	4.76E+01	2.51E+01	5.19E+00
	Rank	1	8	6	9	10	12	3	11	5	7	4	2
cec06	Min	1.80E+03	1.82E+03	1.85E+03	1.97E+03	5.24E+03	4.82E+04	1.98E+03	1.88E+03	2.09E+03	1.81E+03	1.87E+03	1.80E+03
	Max	1.81E+03	5.06E+03	5.69E+03	8.11E+03	2.81E+04	3.58E+06	8.10E+03	8.16E+03	5.26E+04	1.89E+03	7.93E+03	1.80E+03
	Mean	1.80E+03	2.53E+03	3.26E+03	4.34E+03	1.40E+04	1.13E+06	4.28E+03	4.21E+03	1.13E+04	1.83E+03	3.29E+03	**1.80E+03**
	Std	2.66E+00	9.41E+02	1.31E+03	1.82E+03	7.23E+03	8.42E+05	1.96E+03	2.32E+03	1.26E+04	1.79E+01	1.67E+03	5.42E−01
	Rank	2	4	5	9	11	12	8	7	10	3	6	1
cec07	Min	2.02E+03	2.00E+03	2.00E+03	2.02E+03	2.03E+03	2.04E+03	2.00E+03	2.02E+03	2.02E+03	2.00E+03	2.01E+03	2.00E+03
	Max	2.03E+03	2.02E+03	2.06E+03	2.04E+03	2.13E+03	2.07E+03	2.05E+03	2.08E+03	2.06E+03	2.05E+03	2.05E+03	2.02E+03
	Mean	2.03E+03	2.01E+03	2.03E+03	2.03E+03	2.05E+03	2.06E+03	2.02E+03	2.05E+03	2.04E+03	2.01E+03	2.03E+03	**2.01E+03**
	Std	3.67E+00	9.87E+00	1.34E+01	7.37E+00	2.25E+01	6.04E+00	1.01E+01	1.77E+01	1.04E+01	1.17E+01	9.59E+00	6.01E+00
	Rank	6	2	7	8	10	12	4	11	9	3	5	1
cec08	Min	2.21E+03	2.20E+03	2.21E+03	2.22E+03	2.21E+03	2.23E+03	2.22E+03	2.21E+03	2.22E+03	2.20E+03	2.20E+03	2.20E+03
	Max	2.23E+03	2.22E+03	2.22E+03	2.23E+03	2.24E+03	2.37E+03	2.44E+03	2.24E+03	2.35E+03	2.23E+03	2.23E+03	2.22E+03
	Mean	2.22E+03	2.22E+03	2.22E+03	2.23E+03	2.23E+03	2.34E+03	2.25E+03	2.23E+03	2.25E+03	2.22E+03	2.22E+03	**2.21E+03**
	Std	5.35E+00	7.13E+00	3.43E+00	3.03E+00	6.75E+00	4.52E+01	6.11E+01	7.41E+00	4.06E+01	7.30E+00	6.85E+00	4.13E+00
	Rank	2	3	4	7	9	12	11	8	10	6	5	1
cec09	Min	2.41E+03	2.53E+03	2.53E+03	2.53E+03	2.53E+03	2.55E+03	2.53E+03	2.53E+03	2.49E+03	2.53E+03	2.53E+03	2.40E+03
	Max	2.53E+03	2.53E+03	2.53E+03	2.53E+03	2.63E+03	2.78E+03	2.53E+03	2.70E+03	2.71E+03	2.57E+03	2.68E+03	2.53E+03
	Mean	2.52E+03	2.53E+03	2.53E+03	2.53E+03	2.57E+03	2.60E+03	2.53E+03	2.58E+03	2.54E+03	2.54E+03	2.54E+03	**2.52E+03**
	Std	2.57E+01	1.13E−04	1.48E−13	3.42E−04	2.43E+01	5.13E+01	1.49E+00	4.99E+01	8.89E+01	9.29E+00	3.29E+01	2.84E+01
	Rank	2	4	3	5	10	12	6	11	9	7	8	1
cec10	Min	2.50E+03	2.50E+03	2.50E+03	2.41E+03	2.50E+03	2.50E+03	2.50E+03	2.50E+03	2.50E+03	2.50E+03	2.50E+03	2.40E+03
	Max	2.50E+03	2.63E+03	2.65E+03	2.64E+03	2.63E+03	4.11E+03	2.63E+03	2.64E+03	3.39E+03	2.63E+03	2.64E+03	2.50E+03
	Mean	2.50E+03	2.54E+03	2.54E+03	2.56E+03	2.57E+03	2.73E+03	2.55E+03	2.57E+03	2.63E+03	2.55E+03	2.52E+03	**2.43E+03**
	Std	8.88E−02	5.50E+01	6.18E+01	6.93E+01	5.95E+01	4.96E+02	5.75E+01	6.33E+01	2.56E+02	5.96E+01	4.59E+01	2.32E+01
	Rank	2	4	5	8	10	12	6	9	11	7	3	1
cec11	Min	2.60E+03	2.60E+03	2.60E+03	2.60E+03	2.61E+03	3.04E+03	2.60E+03	2.60E+03	2.60E+03	2.60E+03	2.60E+03	2.60E+03
	Max	2.60E+03	2.90E+03	2.90E+03	3.00E+03	2.75E+03	3.90E+03	3.18E+03	3.18E+03	2.90E+03	3.00E+03	2.75E+03	2.60E+03
	Mean	2.60E+03	2.65E+03	2.66E+03	2.75E+03	2.69E+03	3.53E+03	2.65E+03	2.82E+03	2.68E+03	2.67E+03	2.62E+03	**2.60E+03**
	Std	2.41E−01	8.83E+01	1.02E+02	1.60E+02	7.20E+01	2.67E+02	1.54E+02	1.79E+02	1.33E+02	1.15E+02	4.63E+01	2.48E−02
	Rank	2	5	6	10	9	12	4	11	8	7	3	1
cec12	Min	2.86E+03	2.86E+03	2.86E+03	2.86E+03	2.86E+03	2.87E+03	2.86E+03	2.86E+03	2.85E+03	2.87E+03	2.86E+03	2.86E+03
	Max	2.86E+03	2.88E+03	2.88E+03	2.89E+03	2.87E+03	2.93E+03	2.95E+03	2.95E+03	2.90E+03	2.94E+03	2.87E+03	2.86E+03
	Mean	2.86E+03	2.87E+03	2.87E+03	2.87E+03	2.87E+03	2.88E+03	2.89E+03	2.87E+03	2.89E+03	2.88E+03	2.87E+03	**2.86E+03**
	Std	1.31E+00	3.48E+00	3.66E+00	7.53E+00	2.16E+00	1.94E+01	3.35E+01	1.91E+01	2.34E+01	2.12E+01	1.47E+00	1.56E+00
	Rank	2	5	6	7	4	10	12	8	11	9	3	1
Mean ranks		2.83E+00	4.75E+00	5.33E+00	8.08E+00	9.25E+00	1.18E+01	5.50E+00	9.67E+00	8.25E+00	5.92E+00	5.17E+00	1.42E+00
Final ranking		2	3	5	8	10	12	6	11	9	7	4	1

**Table 3:** The *p*-values from the Wilcoxon test across various test functions.

Functions	YDSE	AHA	SO	DO	AO	ChOA	HBA	SCSO	SSA	WSO	COOT
cec01	5.17E−06/-	1.66E−07/-	6.80E−08/-	6.80E−08/-	6.80E−08/-	6.80E−08/-	5.24E−08/-	6.80E−08/-	8.01E−09/-	6.80E−08/-	6.80E−08/-
cec02	7.90E−08/-	1.80E−06/-	2.55E−07/-	1.05E−06/-	6.80E−08/-	6.80E−08/-	3.89E−07/-	6.80E−08/-	6.80E−08/-	2.36E−06/-	1.05E−06/-
cec03	6.80E−08/-	1.60E−05/-	9.05E−03/-	3.50E−06/-	6.80E−08/-	6.80E−08/-	2.47E−04/-	6.80E−08/-	1.06E−07/-	8.10E−02/=	1.41E−05/-
cec04	1.64E−01/=	1.05E−07/-	1.12E−03/-	9.17E−08/-	2.96E−07/-	6.80E−08/-	2.57E−05/-	1.05E−06/-	5.16E−06/-	1.23E−03/-	2.07E−02/-
cec05	7.56E−01/+	1.63E−03/-	4.11E−02/-	1.55E−02/-	6.80E−08/-	6.80E−08/-	6.17E−01/=	6.80E−08/-	1.05E−03/-	2.80E−03/-	8.18E−01/=
cec06	3.99E−06/-	6.80E−08/-	6.80E−08/-	6.80E−08/-	6.80E−08/-	6.80E−08/-	6.80E−08/-	6.80E−08/-	6.80E−08/-	6.80E−08/-	6.80E−08/-
cec07	3.94E−07/-	7.76E−01/=	9.75E−06/-	1.23E−07/-	6.80E−08/-	6.80E−08/-	8.29E−05/-	1.06E−07/-	7.90E−08/-	2.08E−01/=	3.07E−06/-
cec08	1.38E−06/-	5.63E−04/-	1.20E−06/-	6.80E−08/-	1.23E−07/-	6.80E−08/-	1.23E−07/-	6.01E−07/-	6.80E−08/-	5.87E−06/-	8.60E−06/-
cec09	1.94E−08/-	4.63E−05/-	5.74E−01/=	1.95E−08/-	1.95E−08/-	1.95E−08/-	5.02E−03/-	1.95E−08/-	6.52E−02/=	1.95E−08/-	1.69E−04/-
cec10	7.90E−08/-	2.22E−07/-	9.17E−08/-	7.95E−07/-	6.80E−08/-	6.80E−08/-	1.23E−07/-	9.17E−08/-	9.17E−08/-	9.17E−08/-	7.90E−08/-
cec11	9.17E−08/-	3.15E−02/-	3.15E−02/-	2.69E−06/-	6.80E−08/-	6.80E−08/-	6.04E−06/-	6.80E−08/-	3.15E−02/-	6.01E−07/-	5.65E−02/=
cec12	9.89E−01/=	6.80E−08/-	6.80E−08/-	1.06E−07/-	6.80E−08/-	6.80E−08/-	1.43E−07/-	6.80E−08/-	7.04E−03/-	6.80E−08/-	1.23E−07/-
+/=/-	1/2/9	0/1/11	0/1/11	0/0/12	0/0/12	0/0/12	0/1/11	0/0/12	0/1/11	0/2/10	0/2/10

**Table 4:** Comparison of algorithmic complexity between the proposed algorithm and other methods.

Methods	Algorithmic Complexity
YDSE	*O*(*T*_max_×*M*×*n*+*T*_max_×*n*×*d*)
AHA	*O*(*T*_max_×*M*×*n*+*T*_max_×*n*×*d*+*T*_max_×*d/2*)
SO	*O*(*T*_max_×*M*×*n*+*T*_max_×*n*×*d*)
DO	*O*(*T*_max_×*M*×*n*×*d*)
AO	*O*(*T*_max_×*M*×*n*×*d*)
ChOA	*O*((*T*_max_×*n*^2^*/2+T*_max_×*n*×*d/2*)×*M*)
HBA	*O*(*T*_max_×*M*×*n*+*T*_max_×*n*×*d*)
SCSO	*O*(*T*_max_×*M*×*n*×*d*)
SSA	*O*(*T*_max_×*M*×*n*+*T*_max_×*n*×*d*)
WSO	*O*(*T*_max_×*M*×*n*+*T*_max_×*n*×*d*)
COOT	*O*(*T*_max_×*M*×*n*+*T*_max_×*n*×*d*)
FYDSE	*O*(*T*_max_×*M*×*n*+*T*_max_×5*n/2*×*d*)

**Table 5:** Comparison of runtime of the proposed algorithm and other methods in cec2022 suite.

Functions	YDSE	AHA	SO	DO	AO	ChOA	HBA	SCSO	SSA	WSO	COOT	FYDSE
cec01	2.853	4.738	2.732	8.704	6.288	30.353	3.609	15.423	2.001	5.873	2.856	7.785
cec02	2.628	4.432	2.385	8.256	5.765	30.022	3.211	15.111	1.846	5.348	2.445	7.355
cec03	3.496	5.356	3.348	9.227	7.605	31.112	4.079	16.039	2.620	6.109	3.387	10.453
cec04	2.940	4.607	2.673	8.593	6.363	30.290	3.540	15.325	2.047	5.250	2.704	8.306
cec05	3.007	4.753	2.737	8.674	6.556	32.647	3.546	15.435	2.191	5.256	2.799	9.510
cec06	2.722	4.457	2.481	8.383	5.999	36.059	3.252	15.103	1.851	5.068	2.486	7.456
cec07	4.022	5.769	3.765	9.670	8.526	36.019	4.608	16.402	3.139	6.576	3.907	11.638
cec08	4.483	6.147	4.150	10.069	9.489	34.538	4.966	16.934	3.524	6.931	4.191	13.199
cec09	3.750	5.528	3.516	9.352	8.006	31.270	4.347	16.161	2.878	5.616	3.571	10.752
cec10	3.622	5.223	3.348	9.220	7.763	32.908	4.179	15.993	2.796	5.994	3.431	10.250
cec11	4.338	6.132	4.063	10.022	9.253	33.454	4.864	17.164	3.508	6.939	4.144	12.795
cec12	4.519	6.341	4.194	10.102	9.596	32.073	5.040	16.985	3.630	6.455	4.348	13.165

**Table 6:** Probability results of reaching the optimal solution for twenty runs of FYDSE and other methods.

Functions	YDSE	AHA	SO	DO	AO	ChOA	HBA	SCSO	SSA	WSO	COOT	FYDSE
cec01	100.0%	100.0%	95.0%	100.0%	0.0%	0.0%	100.0%	0.0%	100.0%	30.0%	100.0%	100.0%
cec02	55.0%	65.0%	60.0%	35.0%	5.0%	0.0%	25.0%	25.0%	10.0%	65.0%	35.0%	100.0%
cec03	95.0%	95.0%	95.0%	85.0%	5.0%	0.0%	100.0%	10.0%	50.0%	90.0%	80.0%	100.0%
cec04	0.0%	0.0%	10.0%	0.0%	0.0%	0.0%	5.0%	0.0%	5.0%	75.0%	5.0%	20.0%
cec05	100.0%	40.0%	40.0%	45.0%	0.0%	0.0%	80.0%	0.0%	95.0%	45.0%	65.0%	85.0%
cec06	100.0%	0.0%	0.0%	0.0%	0.0%	0.0%	0.0%	0.0%	0.0%	30.0%	0.0%	100.0%
cec07	5.0%	95.0%	10.0%	0.0%	0.0%	0.0%	15.0%	0.0%	0.0%	65.0%	10.0%	85.0%
cec08	70.0%	100.0%	90.0%	10.0%	15.0%	0.0%	55.0%	10.0%	0.0%	15.0%	15.0%	100.0%
cec09	0.0%	0.0%	0.0%	0.0%	0.0%	0.0%	0.0%	0.0%	0.0%	0.0%	0.0%	0.0%
cec10	0.0%	0.0%	0.0%	5.0%	0.0%	0.0%	0.0%	0.0%	0.0%	0.0%	0.0%	45.0%
cec11	100.0%	70.0%	70.0%	45.0%	45.0%	0.0%	90.0%	15.0%	70.0%	60.0%	90.0%	100.0%
cec12	0.0%	0.0%	0.0%	0.0%	0.0%	0.0%	0.0%	0.0%	0.0%	0.0%	0.0%	0.0%
Mean rank	2.917	3.083	4.000	5.083	6.750	7.583	3.250	6.833	4.917	4.083	3.917	1.333

**Table 7:** Results for design variables, gearbox weight, and running time for four-stage gearbox problem.

	FYDSE	YDSE	AHA	SO	DO	AO	ChOA	HBA	SCSO	SSA	WSO	COOT
*x*_1_	15.959	17.016	18.277	18.580	15.968	9.502	14.771	17.945	13.765	23.411	19.509	19.801
*x*_2_	34.100	33.366	32.797	42.315	36.377	17.752	29.781	53.297	24.283	40.983	45.473	45.714
*x*_3_	13.184	14.940	13.963	17.625	15.506	9.088	14.231	23.061	13.055	19.388	18.772	21.534
*x*_4_	29.365	35.591	30.839	35.359	31.706	22.608	31.475	47.149	32.642	41.219	39.499	43.259
*x*_5_	13.417	18.202	16.914	18.354	13.606	9.466	13.568	32.586	13.275	27.790	19.721	26.928
*x*_6_	28.595	32.506	35.106	37.156	30.050	23.479	29.470	48.448	30.596	44.351	38.311	49.284
*x*_7_	14.668	21.524	23.675	21.038	17.118	10.052	14.112	32.269	14.307	24.006	28.694	23.742
*x*_8_	29.226	34.971	39.470	39.596	34.006	28.332	27.777	44.809	31.357	49.318	43.683	46.500
*x*_9_	0.845	2.602	2.953	1.318	1.458	2.802	0.928	1.462	1.254	1.777	1.537	1.448
*x*_10_	0.983	2.589	2.872	1.536	1.715	2.414	0.971	1.421	0.996	1.612	1.787	1.606
*x*_11_	1.013	2.859	2.740	1.253	1.667	2.604	0.911	1.442	1.050	1.604	1.741	1.405
*x*_12_	1.189	2.484	2.329	1.207	1.298	2.435	0.903	1.523	1.268	1.285	1.366	1.533
*x*_13_	2.532	5.957	4.060	4.588	3.145	4.413	2.268	4.529	1.965	4.998	5.143	4.669
*x*_14_	2.859	5.251	5.047	4.755	3.791	4.501	1.187	4.947	2.260	5.988	4.525	5.310
*x*_15_	3.165	5.086	5.402	4.778	3.972	5.297	1.202	5.001	2.557	5.198	5.396	5.142
*x*_16_	2.147	5.116	5.762	4.416	4.526	5.525	1.090	5.017	2.626	4.668	4.759	5.615
*x*_17_	2.384	5.282	4.502	4.687	3.568	4.386	1.162	5.472	2.537	5.085	5.692	5.274
*x*_18_	2.461	4.439	4.817	4.951	3.411	4.061	2.183	5.229	3.001	4.344	4.733	4.306
*x*_19_	2.770	5.563	4.876	4.573	3.394	5.033	1.409	5.246	2.277	5.214	4.398	4.756
*x*_20_	2.248	4.988	4.995	4.259	3.464	4.418	1.514	4.752	3.181	5.060	4.302	4.716
*x*_21_	2.742	4.977	5.937	4.304	4.095	4.750	1.195	4.981	2.496	5.628	4.945	4.941
*x*_22_	2.312	5.366	5.833	4.268	3.672	4.466	0.954	4.414	2.736	4.182	4.476	5.081
Min	14.737	77.356	68.085	47.817	58.589	46.760	107.321	107.255	41.500	44.772	50.437	41.787
Max	98.804	197.889	373.641	135.408	373.641	350.620	143.407	192.523	192.523	280.607	183.075	224.306
Mean	61.861	136.076	203.852	90.482	130.787	126.405	118.456	134.148	106.606	112.777	109.181	97.105
Std	23.355	35.981	90.238	28.931	80.967	76.166	6.806	20.340	41.652	62.520	34.814	43.278
Time	69.382	67.933	71.876	71.277	77.522	141.335	131.252	74.925	97.367	67.870	76.768	69.814

**Table 8:** Optimization restraints for 20-rod truss structures.

Variables	Parameter
Multiple load	Conditions 1: F_1_ is 5 × 10^5^ N, F_2_ is 0, Conditions 2: F_1_ is 0, F_2_ is 5 × 10^5^ N.
Stress	*σ_i_*^max^ is 173.43 MPa.
Displacement constraints	*δ_4y_*^max^ is 60 mm.
Natural frequency constraints	*f*_1_ ≥ 60 Hz and *f*_2_ ≥ 100 Hz.
Continuous cross-sectional area	[*X^low^*^er^, *X*^upper^] is [−100, 100], and critical area is 1 cm^2^.
Material properties	*E* is 6.9 × 10^10^ Pa and *ρ* is 2740 kg/m^3^.

**Table 9:** Optimization results for 20-rod truss structures.

	FYDSE	YDSE	AHA	SO	DO	AO	ChOA	HBA	SCSO	SSA	WSO	COOT
1 (*A*_1_)	1.54E+01	2.43E+01	1.50E+01	1.51E+01	1.52E+01	1.73E+01	3.66E+01	1.52E+01	1.44E+01	1.46E+01	1.81E+01	1.47E+01
2 (*A*_2_)	-	-	-	-	-	2.10E+01	-	-	2.26E+00	-	-	-
3 (*A*_3_)	-	-	-	-	-	-	-	-	-	-	-	-
4 (*A*_4_)	-	-	-	-	-	2.28E+01	3.23E+01	-	-	-	1.28E+01	-
5 (*A*_5_)	2.02E+01	2.38E+01	1.98E+01	1.96E+01	1.95E+01	1.86E+01	-	2.00E+01	1.99E+01	1.99E+01	3.47E+01	2.04E+01
6 (*A*_6_)	-	-	-	-	-	1.92E+01	-	-	8.89E+00	-	1.25E+00	-
7 (*A*_7_)	-	-	-	-	-	-	-	-	-	-	-	-
8 (*A*_8_)	2.03E+01	2.16E+01	2.02E+01	1.96E+01	2.03E+01	1.66E+01	3.36E+01	2.05E+01	1.93E+01	2.05E+01	2.06E+01	2.04E+01
9 (*A*_9_)	-	-	-	-	-	6.06E+00	-	-	1.42E+01	-	-	-
10 (*A*_10_)	-	-	-	-	-	-	-	-	-	-	-	-
11 (*A*_11_)	2.00E+01	2.88E+01	2.00E+01	1.99E+01	2.05E+01	2.42E+01	-	1.97E+01	2.04E+01	2.13E+01	1.49E+01	1.96E+01
12 (*A*_12_)	-	-	-	-	-	-	-	-	8.24E+00	-	-	-
13 (*A*_13_)	2.11E+01	2.16E+01	2.14E+01	2.19E+01	2.13E+01	2.51E+01	3.04E+01	2.13E+01	2.19E+01	2.12E+01	2.23E+01	2.09E+01
14 (*A*_14_)	-	-	-	-	-	-	-	-	-	-	-	-
15 (*A*_15_)	2.10E+01	2.31E+01	2.07E+01	2.13E+01	2.08E+01	1.47E+01	4.65E+01	2.13E+01	2.01E+01	2.13E+01	2.05E+01	2.14E+01
16 (*A*_16_)	-	-	-	-	-	2.06E+01	-	-	-	-	-	-
17 (*A*_17_)	-	-	-	-	-	-	-	-	-	-	-	-
18 (*A*_18_)	3.26E+01	4.33E+01	3.35E+01	3.35E+01	3.30E+01	3.27E+01	4.78E+01	3.27E+01	3.09E+01	3.32E+01	3.25E+01	3.28E+01
19 (*A*_19_)	-	-	-	-	-	1.22E+01	-	-	4.35E+00	-	-	-
20 (*A*_20_)	3.25E+01	3.48E+01	3.26E+01	3.35E+01	3.27E+01	3.26E+01	-	3.29E+01	3.07E+01	3.31E+01	3.25E+01	3.30E+01
Best weight	1.55E+02	1.80E+02	1.55E+02	1.56E+02	1.55E+02	2.28E+02	1.78E+02	1.55E+02	1.82E+02	1.55E+02	1.69E+02	1.55E+02
Mean weight	1.65E+02	1.94E+02	1.76E+02	1.81E+02	1.84E+02	2.61E+02	1.78E+02	1.87E+02	2.11E+02	2.41E+02	2.05E+02	1.80E+02

**Table 10:** Optimization restraints for 24-rod truss structures.

Variables	Parameter
Multiple load	Conditions 1: F_1_ is 5 × 10^4^ N, F_2_ is 0, Conditions 2: F_1_ is 0, F_2_ is 5 × 10^4^ N.
lumped mass on nodes 3	500 kg.
Stress and displacement constraints	*σ_i_*^max^ is 173.43 MPa, *δ*_5*y*&6*y*_^max^ is 10 mm.
Natural frequency constraints	*f*_1_ ≥ 30 Hz.
Continuous cross-sectional area	[*X^low^*^er^, *X*^upper^] is [−40, 40] cm^2^, critical area is 1 cm^2^.
Material properties	*E* is 6.9 × 10^10^ Pa and *ρ* is 2740 kg/m^3^.

**Table 11:** Optimization results for 24-rod truss structures.

	FYDSE	YDSE	AHA	SO	DO	AO	ChOA	HBA	SCSO	SSA	WSO	COOT
1 (*A*_1_)	-	1.32E+01	1.22E+01	-	-	-	-	-	-	-	-	-
2 (*A*_2_)	-	5.13E+00	4.41E+00	-	-	-	-	-	-	-	-	-
3 (*A*_3_)	-	-	-	-	-	-	-	-	-	-	-	-
4 (*A*_4_)	-	-	-	-	-	-	-	-	1.43E+00	-	-	-
5 (*A*_5_)	-	-	-	-	-	-	-	-	-	-	-	-
6 (*A*_6_)	-	1.83E+01	1.08E+01	-	-	-	-	-	2.39E+00	-	-	-
7 (*A*_7_)	2.13E+01	-	-	2.11E+01	2.18E+01	2.85E+01	2.23E+01	2.14E+01	2.15E+01	2.43E+01	1.91E+01	2.12E+01
8 (*A*_8_)	2.66E+00	5.42E+00	1.34E+00	3.12E+00	1.02E+01	2.51E+00	5.36E+00	2.76E+00	3.36E+00	1.45E+01	3.17E+00	3.28E+00
9 (*A*_9_)	-	2.05E+00	-	-	1.63E+00	-	2.23E+00	-	-	-	-	-
10 (*A*_10_)	1.37E+00	-	-	1.34E+00	-	1.47E+01	-	1.41E+00	1.42E+00	1.49E+00	-	1.47E+00
11 (*A*_11_)	-	-	-	-	-	-	-	-	-	-	-	-
12 (*A*_12_)	-	-	-	-	1.28E+01	3.47E+01	-	-	-	-	4.45E+00	-
13 (*A*_13_)	1.81E+01	2.16E+01	1.82E+01	1.86E+01	-	-	2.03E+01	1.79E+01	1.78E+01	1.75E+01	2.05E+01	1.77E+01
14 (*A*_14_)	-	-	-	1.37E+00	1.42E+01	-	-	-	-	-	-	-
15 (*A*_15_)	3.84E+00	7.28E+00	7.08E+00	4.18E+00	4.83E+00	6.86E+00	7.54E+00	4.39E+00	4.09E+00	3.90E+00	4.24E+00	3.84E+00
16 (*A*_16_)	2.42E+01	2.42E+01	2.43E+01	2.42E+01	2.41E+01	2.31E+01	2.43E+01	2.39E+01	2.38E+01	2.44E+01	2.44E+01	2.42E+01
17 (*A*_17_)	-	-	-	-	-	-	-	-	-	-	1.37E+00	-
18 (*A*_18_)	-	-	-	-	-	-	-	-	-	-	-	-
19 (*A*_19_)	-	-	-	-	-	-	-	-	-	-	-	-
20 (*A*_20_)	-	-	-	-	-	-	-	-	-	-	-	-
21 (*A*_21_)	-	-	-	-	-	-	-	-	-	-	-	-
22 (*A*_22_)	-	1.20E+01	-	-	7.22E+00	-	1.38E+00	-	2.26E+00	1.10E+00	-	-
23 (*A*_23_)	-	-	3.09E+00	-	-	-	-	-	-	1.37E+00	3.36E+00	-
24 (*A*_24_)	1.42E+00	7.30E+00	2.50E+00	-	1.04E+00	1.51E+01	1.79E+00	-	1.17E+00	-	-	1.17E+00
Best weight	1.26E+02	1.59E+02	1.31E+02	1.29E+02	1.35E+02	1.62E+02	1.38E+02	1.26E+02	1.36E+02	1.39E+02	1.35E+02	1.26E+02
Mean weight	1.40E+02	1.84E+02	1.50E+02	1.54E+02	1.67E+02	2.03E+02	1.78E+02	1.79E+02	2.06E+02	1.89E+02	2.08E+02	1.47E+02

**Table 12:** Optimization restraints for 72-rod truss structures.

Variables	Parameter
Multiple load conditions	Conditions 1: F_1*x*_ and F_1*y*_ is 22.25 kN, F_1z_ is −22.25 kN, Conditions 2: F_1*z*_, F_2*z*_, F_3*z*_, and F_4*z*_ is −22.25 kN.
Lumped mass on nodes 1, 2, 3, 4	2270 kg.
Stress and displacement constraints	*σ_i_*^max^ is 173.43 MPa, and *δ*^max^_1*x*&1*y*&2*x*&2*y*&3*x*&3*y*&4*x*&4*y*_ is 6.35 mm.
Natural frequency constraints	*f*_1_ ≥ 4 Hz and *f*_3_ ≥ 6 Hz.
Continuous cross-sectional area	[*X^low^*^er^, *X*^upper^] is [−30, 30], and critical area is 1 cm^2^.
Material properties	*E* is 6.9 × 10^10^ Pa and *ρ* is 2767.99 kg/m^3^.

**Table 13:** Optimization results for 72-rod truss structures.

	FYDSE	YDSE	AHA	SO	DO	AO	ChOA	HBA	SCSO	SSA	WSO	COOT
1 (*A*_1_–*A*_4_)	4.88E+00	6.37E+00	5.40E+00	4.60E+00	5.19E+00	7.49E+00	4.96E+00	4.86E+00	5.29E+00	2.54E+01	4.72E+00	6.23E+00
2 (*A*_5_–*A*_12_)	1.09E+01	1.10E+01	8.69E+00	1.12E+01	1.10E+01	8.69E+00	1.22E+01	1.15E+01	1.13E+01	1.21E+01	1.08E+01	1.08E+01
3 (*A*_13_–*A*_16_)	-	-	-	-	-	-	-	-	-	6.33E+00	-	-
4 (*A*_17_–*A*_18_)	-	-	8.79E+00	-	-	9.94E+00	1.00E+01	-	-	-	-	-
5 (*A*_19_–*A*_22_)	9.58E+00	1.09E+01	1.09E+01	1.14E+01	8.16E+00	8.98E+00	1.02E+01	1.05E+01	8.91E+00	2.39E+01	1.14E+01	7.82E+00
6 (*A*_23_–*A*_30_)	8.31E+00	7.88E+00	8.86E+00	7.93E+00	8.03E+00	8.06E+00	8.73E+00	9.33E+00	8.47E+00	1.19E+01	8.47E+00	8.14E+00
7 (*A*_31_–*A*_34_)	-	-	3.07E+00	-	3.23E+00	-	3.87E+00	-	-	-	-	-
8 (*A*_35_–*A*_36_)	3.76E+00	3.69E+00	-	4.00E+00	5.70E+00	-	-	3.51E+00	3.76E+00	-	4.28E+00	4.36E+00
9 (*A*_37_–*A*_40_)	1.08E+01	7.73E+00	1.42E+01	1.13E+01	1.37E+01	9.69E+00	1.25E+01	8.64E+00	1.34E+01	5.48E+01	1.53E+01	1.12E+01
10 (*A*_41_–*A*_48_)	9.42E+00	8.50E+00	7.72E+00	7.77E+00	7.15E+00	7.96E+00	9.26E+00	7.64E+00	8.27E+00	1.33E+01	9.49E+00	7.88E+00
11 (*A*_49_–*A*_52_)	-	-	-	-	-	-	-	-	-	-	-	-
12 (*A*_53_–*A*_54_)	-	-	-	-	-	-	-	-	-	-	-	-
13 (*A*_55_–*A*_58_)	1.54E+01	2.33E+01	1.40E+01	1.46E+01	1.48E+01	1.82E+01	1.89E+01	1.82E+01	1.37E+01	5.74E+01	1.44E+01	1.64E+01
14 (*A*_59_–*A*_66_)	8.35E+00	8.94E+00	6.52E+00	9.50E+00	7.83E+00	8.17E+00	5.68E+00	7.54E+00	8.74E+00	1.20E+01	7.53E+00	9.03E+00
15 (*A*_67_–*A*_70_)	-	-	-	-	-	3.23E+00	-	-	-	-	-	-
16 (*A*_71_–*A*_72_)	-	-	-	-	-	-	-	-	-	-	-	-
Best weight	4.50E+02	4.62E+02	4.55E+02	4.52E+02	4.52E+02	4.57E+02	4.90E+02	4.52E+02	4.50E+02	7.62E+02	4.52E+02	4.50E+02
Mean weight	4.54E+02	4.97E+02	4.80E+02	4.58E+02	4.69E+02	4.68E+02	5.99E+02	4.70E+02	4.91E+02	1.30E+03	1.01E+03	4.60E+02

## Data Availability

All data generated or analyzed during the study are included in this published article.
